# Senescence-related epicardial adipocyte genes lead to immune infiltration and myocardial infarction progression

**DOI:** 10.3389/fcvm.2026.1759091

**Published:** 2026-03-05

**Authors:** Zhihuan Dong, Limin Wang, Yuheng Lang, Ruiying Zhang, Yuchao Wang, Chengxiu Zhao, Qingliang Chen, Yue Zheng

**Affiliations:** 1 Clinical School of Thoracic, Tianjin Medical University, Tianjin, China; 2 Department of Cardiovascular Surgery, Handan First Hospital, Handan, Hebei, China; 3 School of Medicine, Hebei University of Engineering, Handan, Hebei, China; 4 Department of Heart Center, The Third Central Hospital of Tianjin, Tianjin, China; 5 Department of Cardiovascular Surgery, Tianjin Chest Hospital, Tianjin, China

**Keywords:** adipose tissue, CAD, cellular senescence, immune infiltration, myocardial infarction

## Abstract

**Background:**

After coronary artery disease (CAD)-related myocardial injury, reactivation of the epicardium results in cardiac remodeling via paracrine secretion. However, the senescence-related genomic signature that reflects epicardial adipose tissue (EAT) and immune infiltration is not well understood.

**Methods:**

Adipocyte-related differentially expressed genes (DEGs) were identified in EAT and subcutaneous adipose tissue (SAT) from patients with and without CAD. Immune cells and senescence-related DEGs in EATs were identified. A protein-protein interaction network was used to determine the hub genes. To validate these genes, a Gene Expression Omnibus (GEO) dataset investigation, single cell sequencing analysis and the validation of human sub-epicardial adipose and blood samples were performed. To investigate the mechanism, 3T3-L1 cells were used and differentiated to adipocytes and the hub genes were knocked-down and SASPs were determined.

**Results:**

A Venn diagram was used to obtain 82 senescence-related DEGs, and the top 15-degree hub genes were explored. After validating using the GEO datasets and human sub-epicardial adipose samples, *STAT3, SERPINE1, CDKN2A, DLG4, PTGS2, MDM2, LRP1, IRS2, PRKCD, CCND2*, and *CISH* were found to be significantly expressed in the group with severe CAD. The hub genes, including *STAT3, MDM2, LRP1, IRS2, PRKCD, CCND2*, and *CISH*, were validated to be highly expressed in adipocytes in ischemic cardiomyopathy patients with end-stage heart failure. *STAT3, LRP1, PRKCD, CCND2*, and *CISH* were highly expressed in adipocytes under hypoxia. *STAT3, LRP1, PRKCD, CCND2*, and *CISH* were knocked-down in 3T3-L1 cell lines. SASPs, including *IL1α, IL1β,* and *TNFα*, decreased in hypoxic adipocytes after 5 hub genes knockdown. IL6 decreased in hypoxic adipocytes after *STAT3, LRP1,* and *CISH* knockdown*,* while IL6 increased in hypoxic adipocytes after *CCND2* knockdown. The joint ROC of all 5 genes expression was 1.

**Conclusion:**

These screened SASP hub genes, including *STAT3, LRP1, PRKCD, CCND2*, and *CISH,* may affect cell senescence after hypoxia, thus inducing CAD progression.

## 1 Introduction

Coronary artery disease (CAD) continues to be the leading cause of death in low- and middle-income countries (1). The mortality rate of patients with myocardial infarction (MI)-related heart failure (HF) has increased rapidly in the last decade, and obesity is becoming a major cause of CAD (2, 3). As a conservative estimate, 330 million people in China have heart diseases, and young patients with type 2 diabetes mellitus and MI have higher long-term cardiovascular and all-cause mortality, thus requiring more aggressive secondary prevention (2–4).

The effects of adipocytes on cardiomyocytes after MI are greater than previously anticipated. For instance, pericardial adipose tissue regulates granulopoiesis and increases cardiac function after MI (5, 6). Adiponectin, an adipokine, inhibits lipopolysaccharide-induced HMGB1 release in the macrophages (7) and regulates farnesoid X receptor agonism-mediated cardioprotection against post-infarction remodeling and dysfunction (8). In addition, reactivation of the epicardium results in cardiac remodeling after myocardial injury via paracrine secretion (9).

Aging is a phenotype characterized by complex physiological, cellular, and molecular changes (10, 11). Cellular senescence is characterized by stable cell cycle arrest correlated with typical morphological cellular changes and a unique secretome called senescence-associated secretory phenotype (SASP) (12–14). However, the senescence-related genomic signature that reflects epicardial adipose tissue (EAT) and immune infiltration is not well understood.

In this study, adipocyte-related differentially expressed genes (DEGs) were identified, in EAT, subcutaneous adipose tissue (SAT), and pericardial adipose tissue (PAT) from patients with and without CAD. A protein-protein interaction (PPI) network was used to determine the hub genes. Gene Expression Omnibus (GEO) datasets and human blood samples were used to validate the hub genes, which are potential targets of SASPs for diagnosing MI and predicting CAD progression.

## 2 Materials and methods

### 2.1 Data source and data processing

GEO database is a database of gene expression created and maintained by National Center for Biotechnology Information (NCBI). Using the keyword “adipose tissue,” GSE18612 (15) and GSE26339 (16) datasets from the GEO database, NCBI, were downloaded. GSE18612 contains RNA-sequencing data between EAT and SAT from patients with and without CAD. GSE26339 contains RNA-sequencing data between PAT and SAT. The DEGs were explored using the limma package in the Xiantao database (http://www.xiantao.love). An adjusted *P*-value < 0.05 and Log2|FC| > 1 were considered the cut-off criteria.

### 2.2 Pathway enrichment analysis

GO/KEGG pathways were analyzed to explore the functions of DEGs using the Xiantao database (http://www.xiantao.love), a comprehensive website tool for RNA-seq analysis (17, 18). Statistical significance was set at an adjusted *P*-value < 0.05.

### 2.3 PPI and hub genes

To explore genes interactions, the STRING database (https://string-db.org) was used with a combined score > 0.4 (19), and the nodes were analyzed using Cytoscape v.3.7.1 (20). The hub genes were obtained using the Cytoscape plug-in Cytohubba.

### 2.4 Immune infiltration analysis

CIBERSORT can be used to characterize cell abundance in bulk tissues (21). The CIBERSORT algorithm with 100 permutations was applied, with the LM22 matrix used as a reference. Statistical significance was set at an adjusted *P*-value < 0.05. A Pearson correlation analysis was then conducted, and DEGs with Pearson correlation coefficients >0.4 were considered to be immune cell-related.

### 2.5 Senescence-related DEGs in EATs

To investigate the senescence-related DEGs in EAT, the data from GSE35957 were analyzed (22), and those with an adjusted *P*-value < 0.05 and Log2|FC| > 1 were considered significant. A Venn diagram was used to determine overlapping transcripts between the senescence-related and EAT-related DEGs. The PPI network was explored to obtain the hub senescence-related DEGs in EATs.

### 2.6 Hub genes and their interactions

The hub genes and their interactions were investigated using NetworkAnalyst 3.0, which is another comprehensive website tool for RNA-seq analysis and integrates other database resources. Specifically, a transcription factor hub gene network was constructed using the ENCODE ChIP-seq data. Micro RNA-hub gene interactions were determined using miRTarBase v8.0. The hub DEGs-chemicals network was shown using the Comparative Toxicogenomics Database. The hub DEGs-drug network was shown based on the DrugBank database v5.0. GSE139157 data were used to determine hub gene methylation.

### 2.7 Validation using GEO datasets

GSE49937 (23) and GSE775 (24) datasets were downloaded and analyzed using the Xiaotao website tool, which includes RNA-sequencing data on spontaneously developing occlusive–atherosclerotic and operation-induced MI, respectively. Hub gene expression was explored using GSE49937 and GSE775 data, and a *P*value < 0.05 was considered significant. To investigate the effects of the screened genes, a receiver operating characteristic (ROC) curve was constructed, and areas under the curve (AUCs) were determined to explore their diagnostic and prognostic relevance.

### 2.8 Validation using human blood samples

To validate the diagnostic and prognostic relevance of the hub genes, human sub-epicardial adipose tissue samples were collected during surgery and human blood samples were collected at 1d after admission. All experimental protocols were approved by Tianjin Third Central Hospital. All methods were carried out in accordance with relevant guidelines and regulations. Informed consent was obtained from all subjects or their legal guardian.

The cohorts with CAD were divided into two groups: the single vessel lesion group (CAD group) and the multi-vessel lesion group (severe CAD group). Participants were included according to their baseline characteristics, such as sex, age, and prior medical history. The inclusion criteria were as follows: (1) patients undergoing coronary artery bypass graft surgery; (2) patients whose sub-epicardial adipose tissue samples could be collected; and (3) patients with complete information and laboratory examination data. Patients with a history of any of the following were excluded: (1) nephropathy, hepatopathy, diabetic oculopathy, brain damage, or tumor; (2) cardiac arrest or extracorporeal cardiopulmonary resuscitation; (3) aortic insufficiency or aortic dissection; or (4) uncontrolled bleeding.

The hub genes were analyzed using human subepicardial adipose tissue samples. The hub genes protein expressions were analyzed using human blood samples. Clinical information and laboratory examination results were also collected.

### 2.9 Single-cell sequencing data validation

Single-cell sequencing data were obtained from the Single Cell Portal (https://singlecell.broadinstitute.org/) to explore the hub gene expression in different cell lines (SCP1849). SCP1849 was the single-cell sequencing data about human ischemic cardiomyopathy patients with end-stage heart failure.

### 2.10 Cell source and processing

3T3-L1 (fibroblast) cell lines were purchased from Chinese Academy of Medical Sciences and was cultured in DMEM medium containing 10% (v/v) fetal bovine serum (FBS) in a 37 ℃, 5% CO2 incubator before experiment.

The hub genes expressions in 3T3-L1 cells were knockdown by adenovirus (m-hub genes-shRNA-GFP-Puro from 293 T cells, 6.57 × 108 TU/mL, about MOI:100). Cells were cultured in 6-well plates (5 × 10^5^ cells per well), transfected using shRNA adenovirus (MOI = 10), and screened using puromycin (2 μg/mL, Genechem, Shanghai) to obtain the infected 3T3-L1 cells. Adipocyte differentiation was induced as previously described using 3T3-L1 cells (25). sh Scramble was used as a negative control.

### 2.11 qPCR analysis

qRT-PCR was performed using TRIzol and TB Green method (TaKaRa, RR820). *β*-actin was the reference gene and the 2-*ΔΔ*Ct method was utilized. The knockdown efficiency was determined using qRT-PCR. The hub genes mRNA expressions were analyzed in adipocytes under hypoxia using qRT-PCR. The knockdown efficiency was also measured using qRT-PCR. The primer details were shown in Supplementary Table S1.

### 2.12 ELISA

Human STAT3 ELISA Kit (LS-F819-1, LSBio), LRP1 ELISA Kit (LS-F55616, LSBio), PRKCD ELISA Kit (USEA433Hu, Cloud-Clon Corp.), CCND2 ELISA Kit (LS-F1926, LSBio) and CISH ELISA Kit (LS-F18300, LSBio) were used to determine the expressions in the human blood samples of HFpEF patients and HFrEF patients.

Mouse IL-1*α* ELISA Kit (EK201A, MULTI SCIENCES), mouse IL-1β ELISA Kit (EK201BHS, MULTI SCIENCES), mouse IL-6 ELISA Kit (PI326, Beyotime) and mouse TNF*α* ELISA Kit (PT512, Beyotime) were used to determine the expressions in the cell culture supernatants to analyze the SASPs and cell senescence.

### 2.13 Statistical analysis

Data are presented as the mean ± standard deviation (SD), median (Q1–Q3), or frequency (percentage). Statistical analyses were performed using SPSS version 23.0. The Shapiro–Wilk normality test and Welch's *t*-test (two groups) were conducted. Statistical significance was set at *P* < 0.05.

## 3 Results

### 3.1 Identification of DEGs in EAT and further analyses

Thirty-five up-regulated and 122 down-regulated DEGs were obtained between the EAT of patients with and without CAD from the GSE18612 dataset that were mainly involved in the site of polarized growth and the growth cone (Supplementary Figures S1A, D; Table S2). In patients with CAD, 222 up-regulated and 188 down-regulated DEGs were obtained between the EAT and SAT that were mainly involved in the negative regulation of anoikis, endocrine resistance, and focal adhesion (Supplementary Figures S1B, E; Table S3). In patients without CAD, 14 up-regulated and 227 down-regulated DEGs were obtained between the EAT and SAT that were mainly involved in the microtubule organizing center, AMPK signaling pathway, longevity regulating pathway, and negative regulation of actin filament polymerization (Supplementary Figures S1C and S2; Table S4).

The STRING database and Cytoscape plug-in Cytohubba were utilized to investigate the hub genes of EAT. The top 15-degree hub genes between the EAT (including *F2*, *DLG4*, and *LRP1*) of patients with and without CAD were identified (Supplementary Figures S1F, G). The top 15-degree hub genes between the EAT and SAT of patients with CAD were also identified, including *IGF1*, *PTK2*, *CDKN2A*, *STAT1*, and *JAK2* (Supplementary Figures S1H, I).

### 3.2 Identification of DEGs in PAT and further analyses

A total of 190 up-regulated and 149 down-regulated DEGs in PAT were also obtained between the whole PAT and SAT that were mainly involved in leukocyte migration, chemokine-mediated signaling pathways, collagen-containing extracellular matrix, and primary immunodeficiency. A total of 115 up-regulated and 53 down-regulated DEGs were obtained between the isolated PAT and SAT that were mainly involved in the regulation of immune effector processes and lymphocyte activation, membrane rafts, and complement and coagulation cascades. The transcripts between the whole- and isolated-tissue related DEGs were identical for 95 of the obtained DEGs (Supplementary Figures S3A-D; Table S5, S6). Using the STRING and Cytoscape databases, the top 30-degree hub genes were identified in whole DEGs, including *PAX5*, *CCL2*, *PTGS2*, *APOE*, *LCK*, and *TBX21* (Supplementary Figures S3E, F). According to the immune infiltration analysis of whole PAT-related DEGs, the abundance of M0 macrophages, CD4 + naïve T cells, and mast cells were significantly different between PAT and SAT. Additionally, immune cell-related DEGs, which could be associated with immune cells that are stored in the pericardial layer and respond to inflammation under cardiac stress (e.g., after CAD), were also obtained (Supplementary Figures S3G, H; Table S7).

### 3.3 Senescence-related DEGs and further analyses

**Figure 1:**
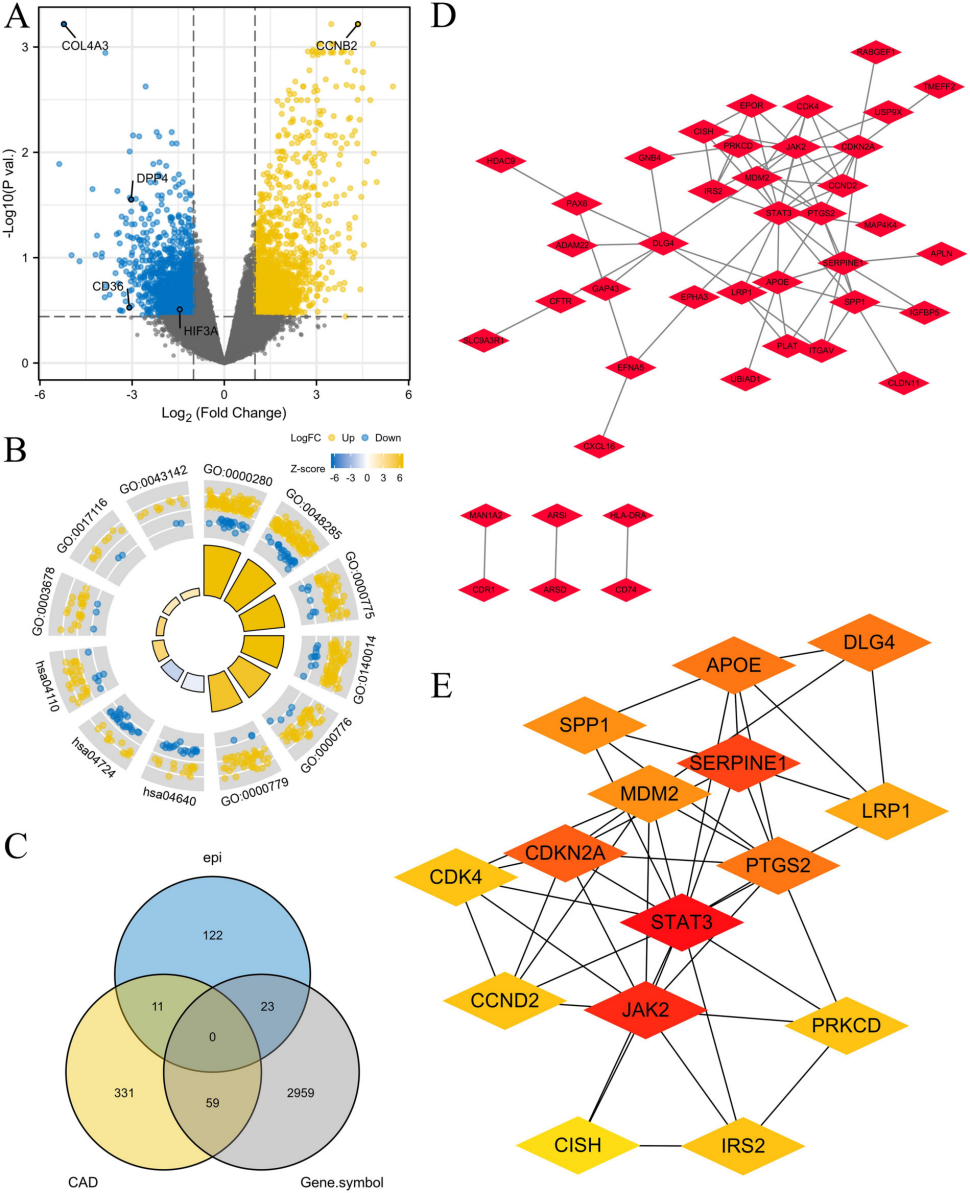
The senescence-related DEGs in EAT. **(A)** The volcano plot of the senescence-related DEGs in MSCs. **(B)** The GO/KEGG pathways enriched by the senescence-related DEGs. **(C)** Venn diagram of the senescence-related DEGs, DEGs between EAT in CAD and non-CAD subjects (epi) and DEGs between EAT and SAT in CAD patients (CAD). **(D,E)** The PPI network **(D)** and the hub genes **(E)** were obtained from the screened 82 genes.

To investigate the effects of hub genes on cell senescence, EAT phentypes and MI progression, Senescence-related DEGs in EAT were obtained and mainly involved in mitotic nuclear division, the hematopoietic cell lineage, and the cell cycle (Figure 1A, B; Table 1). A Venn diagram was used to obtain the same transcripts, and the PPI network was used for 82 overlapping genes. The top 15-degree hub genes were identified, including *JAK2*, *STAT3*, *LRP1*, *PTGS2*, *CDKN2A*, and *SPP1* (Figures 1C–E; Table 2).

**Table 1:** The GO/KEGG pathways enriched by senescence related DEGs in MSCs.

Ontology	ID	Description	GeneRatio	BgRatio	pvalue	p.adjust	qvalue
BP	GO:0000280	nuclear division	113/2215	407/18670	1.06e-18	6.53e-15	5.34e-15
BP	GO:0048285	organelle fission	119/2215	449/18670	6.72e-18	2.06e-14	1.69e-14
BP	GO:0140014	mitotic nuclear division	79/2215	264/18670	2.07e-15	4.23e-12	3.46e-12
CC	GO:0000775	chromosome, centromeric region	64/2316	193/19717	2.88e-15	1.98e-12	1.70e-12
CC	GO:0000776	kinetochore	49/2316	135/19717	1.04e-13	3.08e-11	2.63e-11
CC	GO:0000779	condensed chromosome, centromeric region	45/2316	118/19717	1.34e-13	3.08e-11	2.63e-11
MF	GO:0003678	DNA helicase activity	26/2216	81/17697	3.28e-06	0.002	0.002
MF	GO:0017116	single-stranded DNA-dependent ATP-dependent DNA helicase activity	11/2216	20/17697	6.57e-06	0.002	0.002
MF	GO:0043142	single-stranded DNA-dependent ATPase activity	11/2216	20/17697	6.57e-06	0.002	0.002
KEGG	hsa04640	Hematopoietic cell lineage	34/1034	99/8076	2.47e-08	7.92e-06	6.57e-06
KEGG	hsa04724	Glutamatergic synapse	35/1034	114/8076	3.64e-07	5.84e-05	4.84e-05
KEGG	hsa04110	Cell cycle	36/1034	124/8076	1.13e-06	1.20e-04	9.99e-05

DEGs, different expressed genes; MSCs, mesenchymal stem cells; GO, gene ontology; BP, biological process; CC, cellular component; MF, molecular function; KEGG, Kyoto Encyclopedia of Genes and Genomes.

**Table 2:** The details of the hub genes.

Gene Symbol	Full name	Functions
STAT3	Signal transducer and activator of transcription 3	Signal transducer and transcription activator that mediates cellular responses to interleukins, KITLG/SCF, LEP and other growth factors.
JAK2	Tyrosine-protein kinase JAK2	Non-receptor tyrosine kinase involved in various processes such as cell growth, development, differentiation or histone modifications. Mediates essential signaling events in both innate and adaptive immunity.
SERPINE1	Plasminogen activator inhibitor 1	Serine protease inhibitor. This inhibitor acts as “bait” for tissue plasminogen activator, urokinase, protein C and matriptase-3/TMPRSS7.
CDKN2A	Cyclin-dependent kinase inhibitor 2A	Acts as a negative regulator of the proliferation of normal cells by interacting strongly with CDK4 and CDK6. This inhibits their ability to interact with cyclins D and to phosphorylate the retinoblastoma protein
DLG4	Disks large homolog 4	Interacts with the cytoplasmic tail of NMDA receptor subunits and shaker-type potassium channels. Required for synaptic plasticity associated with NMDA receptor signaling.
APOE	Apolipoprotein E	Mediates the binding, internalization, and catabolism of lipoprotein particles.
PTGS2	Prostaglandin G/H synthase 2	Converts arachidonate to prostaglandin H2 (PGH2), a committed step in prostanoid synthesis. Constitutively expressed in some tissues in physiological conditions, such as the endothelium, kidney and brain, and in pathological conditions.
SPP1	Secreted phosphoprotein 1	Osteopontin; Binds tightly to hydroxyapatite. Appears to form an integral part of the mineralized matrix.
MDM2	E3 ubiquitin-protein ligase Mdm2	E3 ubiquitin-protein ligase that mediates ubiquitination of p53/TP53, leading to its degradation by the proteasome. Inhibits p53/TP53- and p73/TP73-mediated cell cycle arrest and apoptosis by binding its transcriptional activation domain.
LRP1	Prolow-density lipoprotein receptor-related protein 1	Endocytic receptor involved in endocytosis and in phagocytosis of apoptotic cells. Required for early embryonic development. Involved in cellular lipid homeostasis.
IRS2	Insulin receptor substrate 2	mediate the control of various cellular processes by insulin; Pleckstrin homology domain containing
PRKCD	Protein kinase C delta type	Calcium-independent, phospholipid- and diacylglycerol (DAG)-dependent serine/threonine-protein kinase that plays contrasting roles in cell death and cell survival by functioning as a pro-apoptotic protein during DNA damage-induced apoptosis.
CCND2	G1/S-specific cyclin-D2	Regulatory component of the cyclin D2-CDK4 (DC) complex that phosphorylates and inhibits members of the retinoblastoma (RB) protein family including RB1 and regulates the cell-cycle during G(1)/S transition.
CDK4	Cyclin-dependent kinase 4	Ser/Thr-kinase component of cyclin D-CDK4 (DC) complexes that phosphorylate and inhibit members of the retinoblastoma (RB) protein family including RB1 and regulate the cell-cycle during G(1)/S transition.
CISH	Cytokine-inducible SH2-containing protein	SOCS family proteins form part of a classical negative feedback system that regulates cytokine signal transduction. CIS is involved in the negative regulation of cytokines that signal through the JAK-STAT5 pathway such as erythropoietin, prolactin and interleukin 3 (IL3) receptor.

### 3.4 Hub genes and their interactions

**Figure 2:**
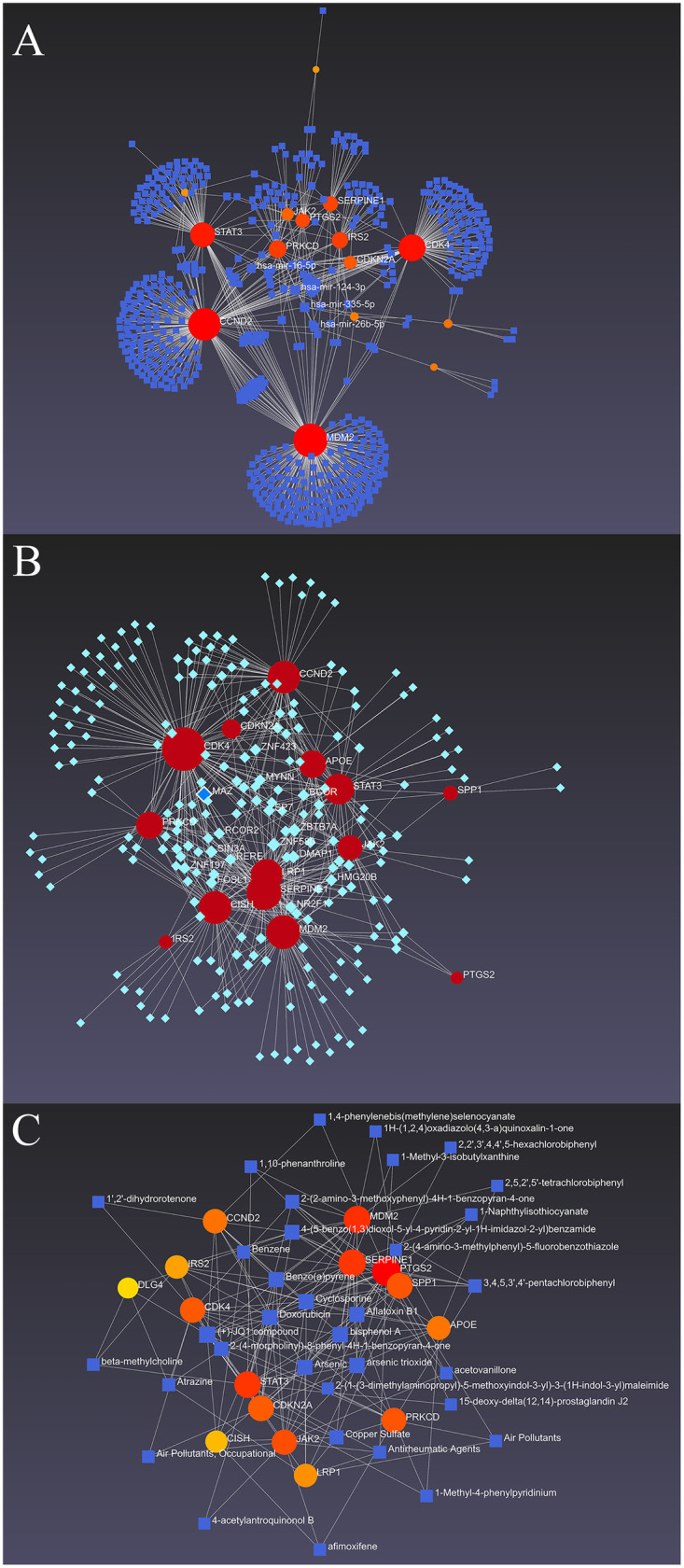
The hub genes and their interactions. The networks of miRNA-the hub DEGs **(A)**, TFs- the hub DEGs **(B)** and chemicals- the hub DEGs **(C)** were constructed.

The screened DEGs and their interactions are shown in Figure 2 and S4. Transcription factors, micro RNAs, drugs, and chemical network interactions with hub DEGs were determined to provide further insight into the treatment of aging patients with CAD. Methylation of the 82 screened overlapping genes was also investigated, demonstrating high methylation of *CDKN2A* and *CCND2* and low methylation of *DLG4* and *LRP1* (Supplementary Figure S5; Table S8).

### 3.5 Validation of the hub genes using GEO datasets

**Figure 3:**
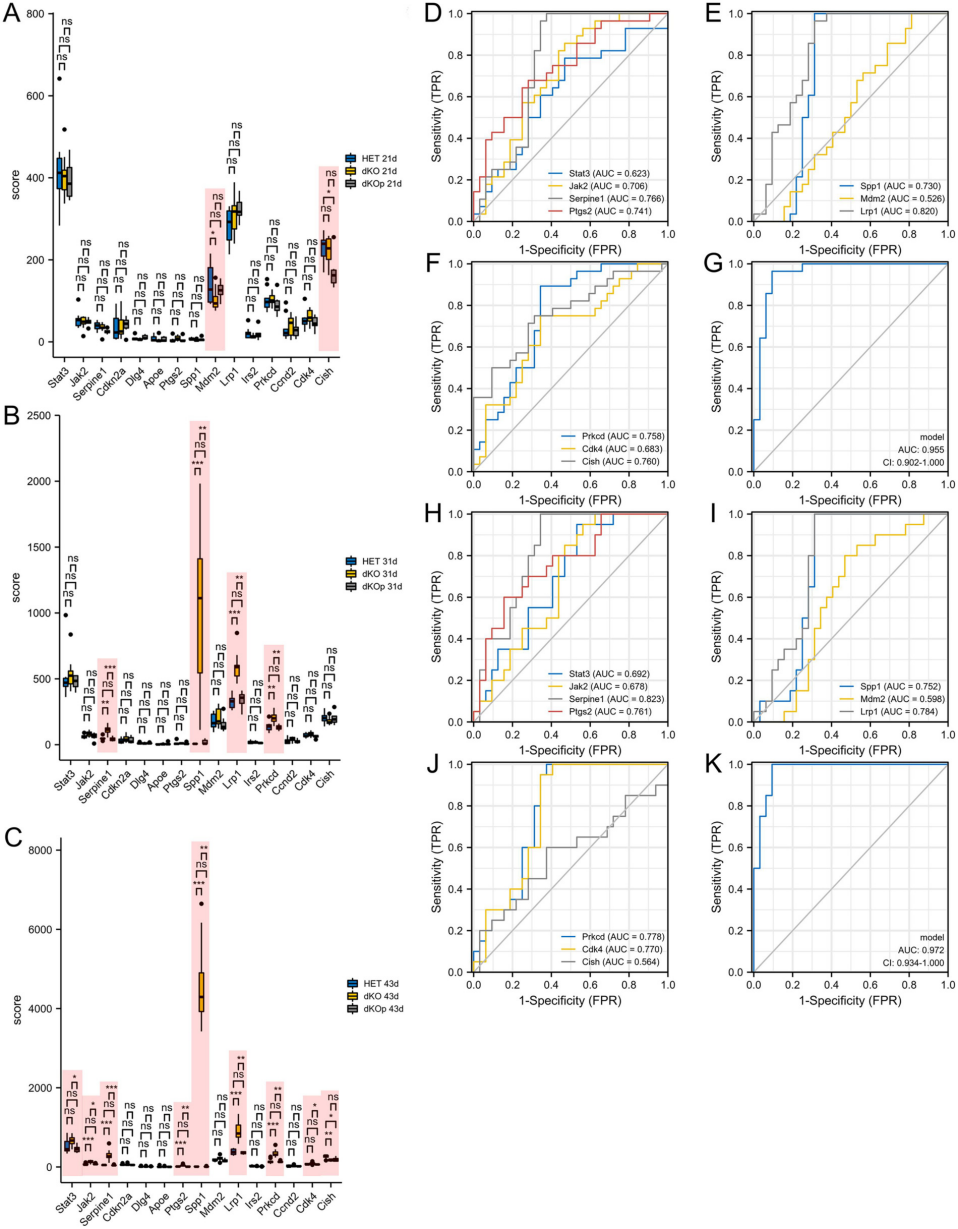
The hub genes expressions validation in subcutaneous MI mice model. **(A–C)** The hub genes expressions were validated in 21d **(A)**, 31d **(B)** and 43d **(C)** after birth in dKO mice group, heterozygote group and dKO treatment group. **(D–G)** The ROCs **(D–F)** and the joint ROC **(G)** were used to explore the diagnostic relevance of the hub genes. **(H–K)** The ROCs **(H–J)** and the joint ROC **(K)** were used to explore the prognostic values of the hub genes. *P < 0.05; **P < 0.01; ***P < 0.001; ns, not significant.

The expression of the hub genes in the GSE49937 dataset was determined to investigate their effects on subcutaneous MI (Figures 3A–C). *SERPINE1*, *SPP1*, *LRP1*, and *PRKCD* were significantly highly expressed within 31 days of birth in the dKO mice group compared to the heterozygote group, and after treatment, the expression levels were rescued. *JAK2, SERPINE1, SPP1, PTGS2, LRP1,* and *PRKCD* were highly expressed at 43 days in the dKO mice group, and after treatment, the expression levels were rescued. ROC curves were used to investigate the diagnostic relevance of the hub genes. The AUCs of *JAK2*, *SERPINE1*, *SPP1*, *PTGS2*, *LRP1*, *CISH*, and *PRKCD* were over 0.7 each, and the combined AUC was 0.955, suggesting a high diagnostic relevance for subcutaneous MI progression (Figures 3D–G). ROC curves were also used to investigate the prognostic value of the hub genes. The AUCs of *SERPINE1*, *SPP1*, *PTGS2*, *LRP1*, *CDK4*, and *PRKCD* were over 0.7 each, and the combined AUC was 0.972, suggesting high prognostic value for subcutaneous MI (Figures 3H–K).

**Figure 4:**
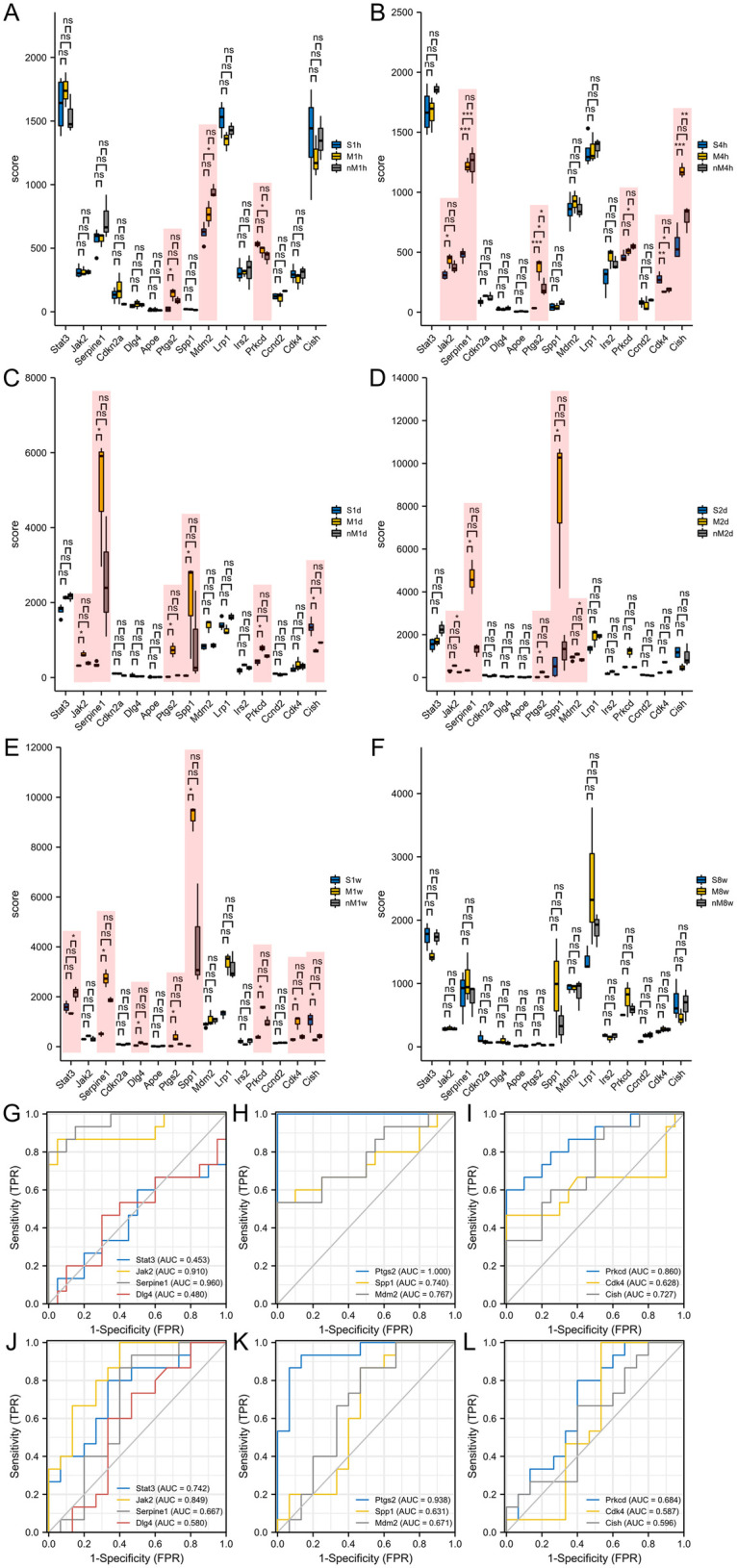
The hub genes expressions validation in operation-induced MI mice model. **(A–F)** The hub genes expressions were validated in 1 h **(A)**, 4 h **(B)**, 1d **(C)**, 2d **(D)**, 1w **(E)** and 8w **(F)** after MI. **(G–I)** The ROCs were used to explore the diagnostic relevance of the hub genes. **(J–L)** The ROCs were used to explore the prognostic values of the hub genes. *P < 0.05; **P < 0.01; ***P < 0.001; ns, not significant.

The expression of the hub genes in the GSE775 dataset was determined to investigate their effects on subcutaneous MI (Figures 4A–F). *JAK2*, *SERPINE1*, *SPP1*, *PTGS2*, *LRP1*, *CDK4*, *CISH*, and *PRKCD* were significantly expressed within 3 days of the MI, whereas *STAT3*, *SERPINE1*, *SPP1*, *PTGS2*, *DLG4*, *CDK4*, *CISH*, and *PRKCD* were significantly expressed 1 week after MI. In addition, no significant differences in the hub genes at 8 weeks post-MI were observed. ROC curves were used to investigate the diagnostic relevance of the hub genes. The AUCs of *JAK2*, *SERPINE1*, *PTGS2*, and *PRKCD* were 0.910, 0.960, 1, and 0.860, respectively, suggesting high diagnostic relevance for MI progression (Figures 4G–I). Additionally, the joint ROC of all gene expression between CAD and non-CAD patients was 1 (Supplementary Figure S6). ROC curves were also used to investigate the prognostic value of the hub genes. The AUCs of *STAT3*, *JAK2*, and *PTGS2* were 0.742, 0.849, and 0.938, respectively, suggesting high prognostic value for MI (Figures 4J–L).

### 3.6 Validation of the Hub genes using human samples and cell lines

**Figure 5:**
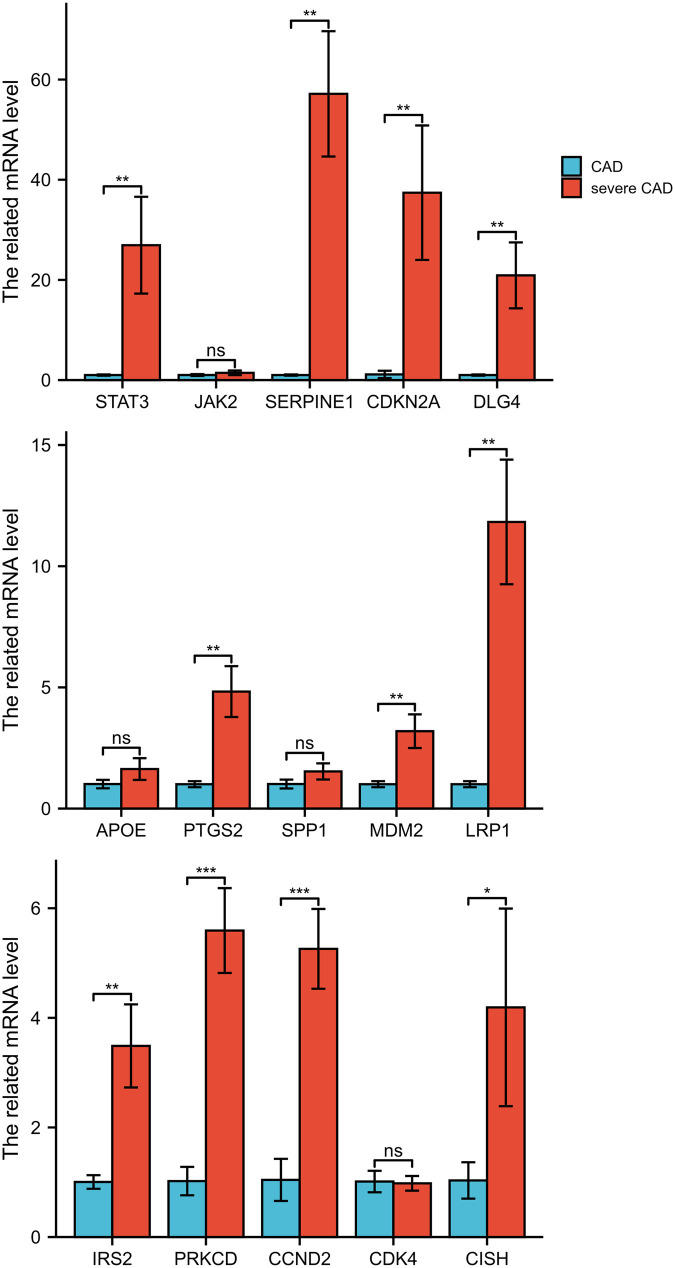
The 15 hub genes expression in human adipose tissues from patients with CAD and severe CAD. *P < 0.05; **P < 0.01; ***P < 0.001; ns, not significant. *N* = 3 each group.

No significant difference in baseline characteristics and laboratory examinations 1 day after admission between the CAD and severe CAD groups was observed (Supplementary Tables S9 and S10). After surgery, fasting venous blood glucose and total bilirubin levels were still higher in the severe CAD group than in the CAD group (Supplementary Table S11). Using qPCR analysis of adipose tissues in the sub-myocardial layer, *STAT3*, *SERPINE1*, *CDKN2A*, *DLG4*, *PTGS2*, *MDM2*, *LRP1*, *IRS2*, *PRKCD*, *CCND2*, and *CISH* were found to be highly expressed in the severe CAD group (Figure 5), suggesting they may be potential prognostic biomarkers for MI progression and recurrence.

**Figure 6:**
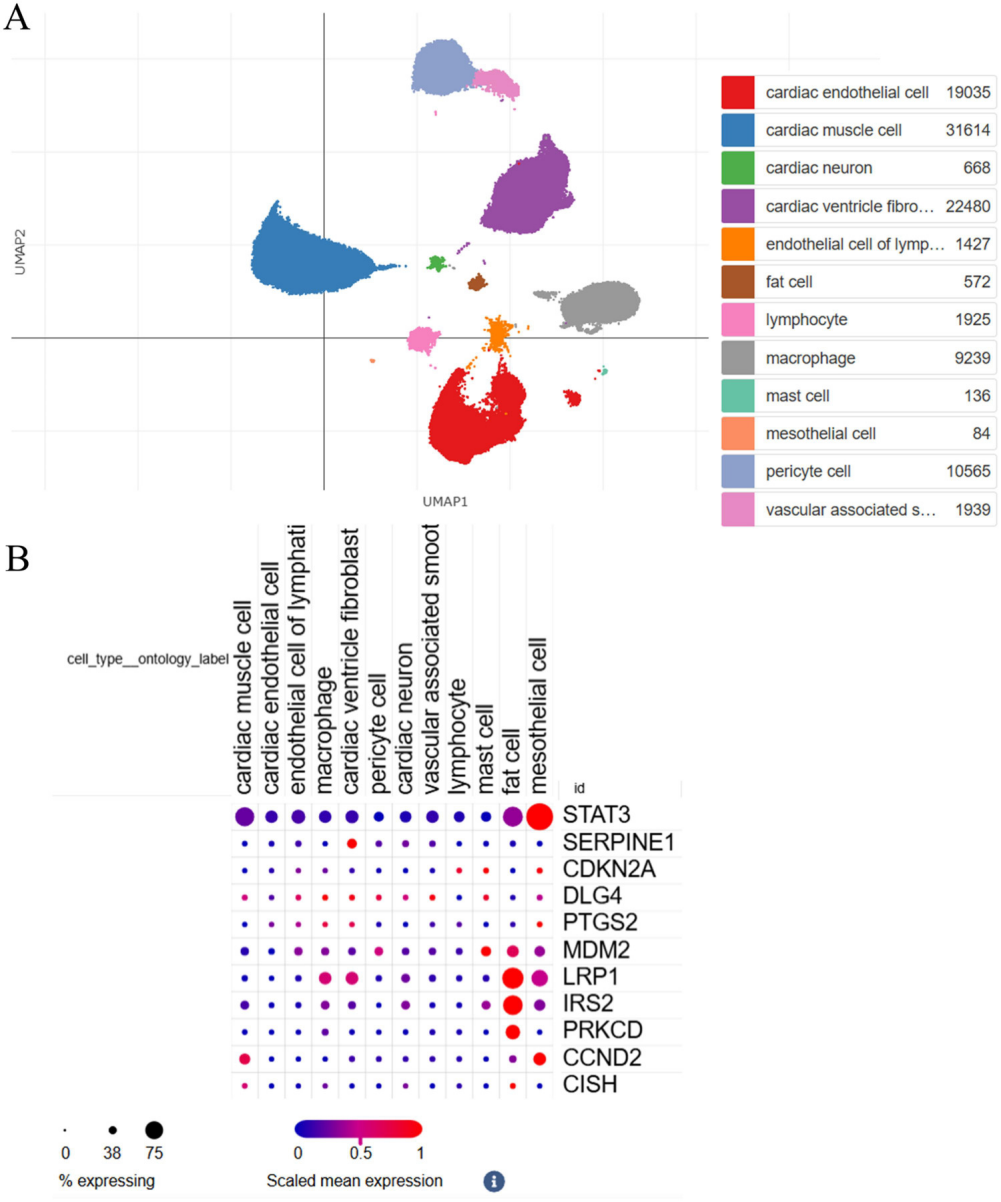
The hub DEGs were validated in ischemic cardiomyopathy patients with end-stage heart failure using single-cell sequencing data. **(A)** The overall clustering of cells in ischemic cardiomyopathy patients with end-stage heart failure. **(B)** The hub genes were validated to be highly expressed in adipocytes in ischemic cardiomyopathy patients with end-stage heart failure, including STAT3, MDM2, LRP1, IRS2, PRKCD, CCND2 and CISH.

**Figure 7:**
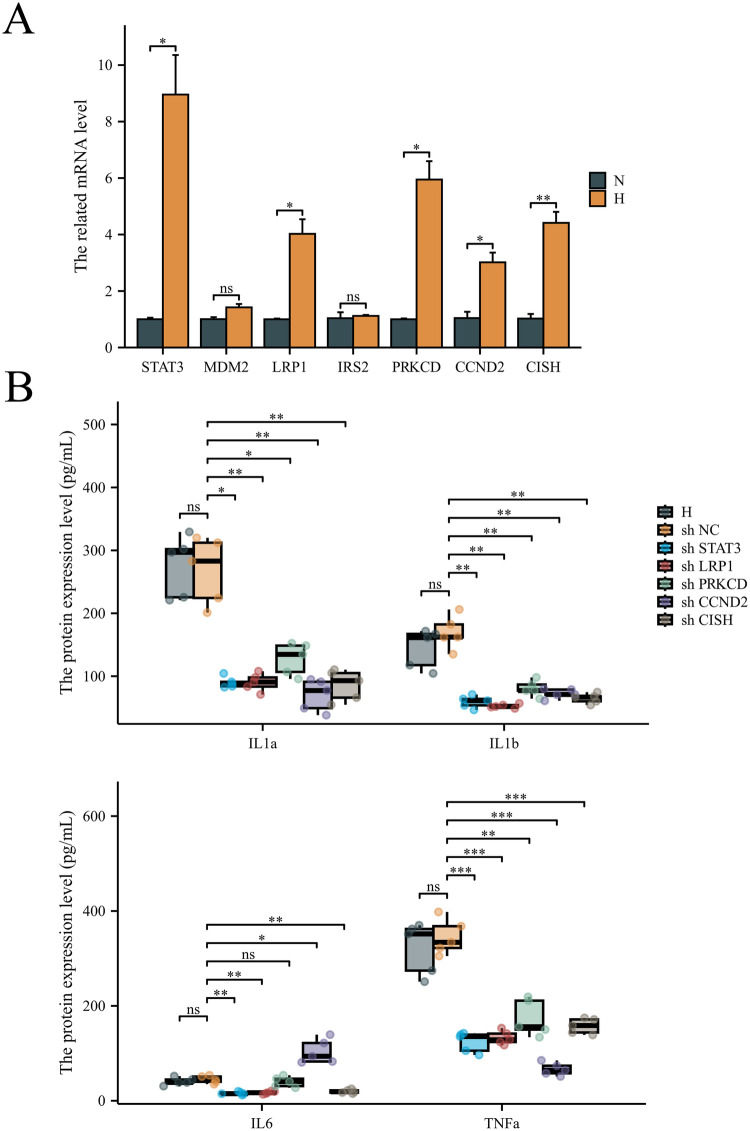
The effects of the hub genes on adipocytes senescence. **(A)** The hub genes related mRNA level in adipocytes under hypoxia or normal. *N* = 3 each group. **(B)** ELISA was performed to determine the SASPs in adipocytes under hypoxia and siRNA knockdown. *n* = 6 per group. *P < 0.05; **P < 0.01; ***P < 0.001; ns, not significant.

**Figure 8:**
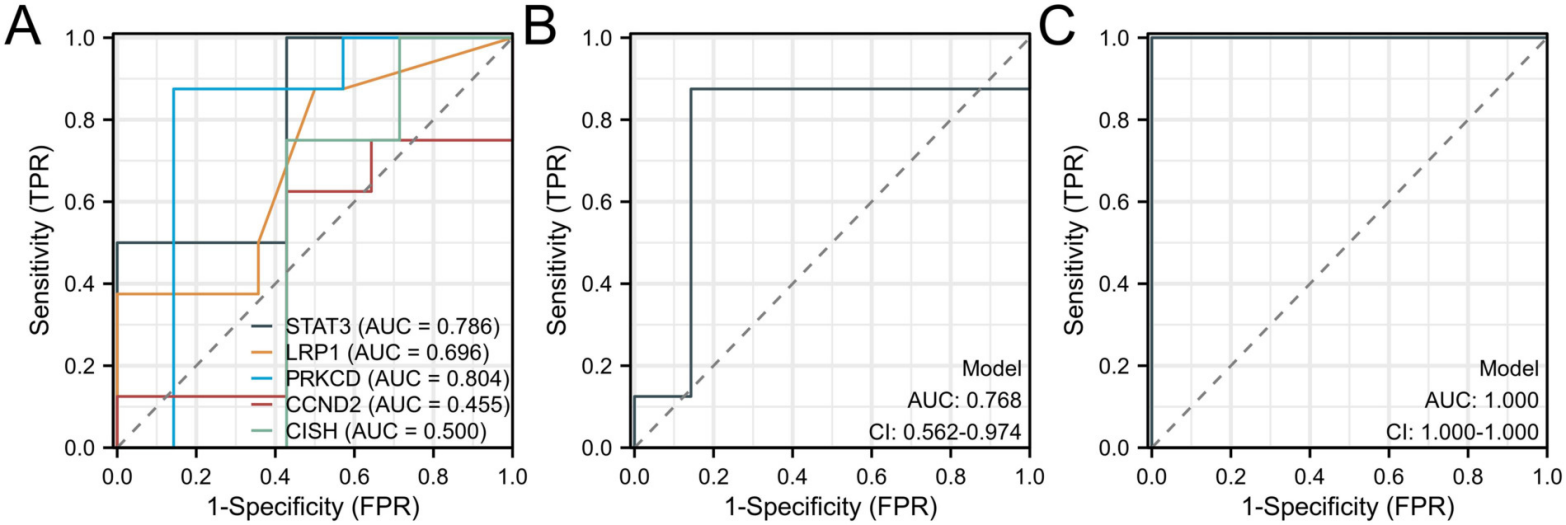
ROC analysis was performed in human blood samples to investigate the diagnostic relevance between patients with CAD and severe CAD. **(A)** ROC of STAT3, LRP1, PRKCD, CCND2 and CISH expression between patients with CAD and severe CAD. **(B)** The joint ROC of STAT3, LRP1 and PRKCD expression between patients with CAD and severe CAD. **(C)** The joint ROC of all 5 genes expression between patients with CAD and severe CAD. *n* = 232 in CAD group and *n* = 132 in severe CAD group.

Then, the hub DEGs were validated in ischemic cardiomyopathy patients with end-stage heart failure using single-cell sequencing data (Figure 6). The hub genes, including *STAT3, MDM2, LRP1, IRS2, PRKCD, CCND2*, and *CISH*, were validated to be highly expressed in adipocytes in ischemic cardiomyopathy patients with end-stage heart failure. To investigate the effects of *STAT3, MDM2, LRP1, IRS2, PRKCD, CCND2*, and *CISH* expression on adipocytes in CAD progression, 3T3-L1 cell lines were utilized and differentiated to adipocytes. *STAT3, LRP1, PRKCD, CCND2*, and *CISH* were highly expressed in adipocytes under hypoxia (Figure 7A). *STAT3, LRP1, PRKCD, CCND2*, and *CISH* were knocked-down in 3T3-L1 cell lines (Supplementary Figure S7). SASPs, including *IL1α, IL1β,* and *TNFα*, decreased in hypoxic adipocytes after 5 hub genes knockdown. IL6 decreased in hypoxic adipocytes after *STAT3, LRP1,* and *CISH* knockdown*,* while IL6 increased in hypoxic adipocytes after *CCND2* knockdown (Figure 7B). Also, we determined the diagnostic relevance of 5 hub genes. The ROC of *STAT3, LRP1, PRKCD, CCND2*, and *CISH* expression between patients with CAD and severe CAD were 0.786, 0.696, 0.804, 0.455 and 0.500, respectively (Figure 8A). The joint ROC of *STAT3, LRP1* and *PRKCD* (ROC >0.6) expression between patients with CAD and severe CAD was 0.768, while the joint ROC of all 5 genes expression was 1 (Figures 8B,C). These results suggested that *STAT3, LRP1, PRKCD, CCND2*, and *CISH* may affect cell senescence after hypoxia, thus inducing CAD progression.

## 4 Discussion

The etiology of stable CAD and MI is relatively clear; however, clinical treatment targets remain limited (26). The occurrence of MI is positively influenced by plaque progression and monocyte and macrophage infiltration (4, 27, 28). M2-like macrophages regulate Nrg1/ErbB signaling during fibrotic tissue formation after MI, which suppresses cardiac fibroblast senescence (29). Macrophage migration inhibitors rejuvenate aged mesenchymal stem cells in humans, thus improving myocardial repair after MI (30). In this study, 35 up-regulated and 122 down-regulated DEGs were obtained between the EAT of patients with and without CAD, and a Venn diagram was used to determine which ones had the same transcript (82 overlapping genes). A Venn diagram was used to obtain the same transcripts between senescence-related and EAT-related DEGs, and the PPI network was used for 82 overlapping genes. The top 15-degree hub genes were identified, including *JAK2*, *STAT3*, *LRP1*, *PTGS2*, *CDKN2A*, and *SPP1*, which may be potential targets of SASPs for predicting MI progression and recurrence.

Aging is considered to be the major risk factor for the development of atherosclerosis and, therefore, for CAD. Aging contributes to lipoprotein metabolism disorders in humans and animals, including the changes in the liver sinusoidal endothelium, postprandial lipemia, insulin resistance induced by free fatty acid (FFA), and activity of peroxisome proliferator-activated receptor *α* (PPAR*α*) (31). Chronologic age is the dominant risk factor for CAD and genomic aging, such as *DNMT3A, TET2, ASXL1,* and *JAK2* mutations, is associated with CAD progression (32). Besides, aging is also associated with a reduction in the regenerative capacity of the endothelium and endothelial senescence, for instance, the activity of the endothelial NO synthase, upregulation of the inducible NO synthase and increased formation of reactive oxygen species, which is characterized by an increased rate of endothelial cell apoptosis (33).

After validation using GEO datasets and human sub-myocardial adipose tissue samples, 13 hub genes, including *STAT3*, *SERPINE1*, *CDKN2A*, *DLG4*, *PTGS2*, *MDM2*, *LRP1*, *IRS2*, *PRKCD*, *CCND2*, and *CISH*, were highly expressed in the severe CAD group; thus, these may be potential prognostic biomarkers for MI progression and recurrence. Adipose-derived stem cells impede cardiac remodeling by regulating macrophage polarization through the PI3 K/STAT3 pathway after MI (34). Leptin promotes the binding of phosphorylated-STAT3 to the *PTGS2* promoter in cardiac myocytes (35) and macrophages (36). *SPP1*, *SERPINE1*, *PTGS2*, and *IL-6* are dependable molecular biomarkers for screening, diagnosing, and determining the prognosis of MI and related to ferroptosis and autophagy phenotype (26, 37), which is consistent with our results. The *PTGS2* variant rs20417 polymorphism decreases the risk of MI recurrence (38). In a hypercholesterolemic mouse model (B6-Ldlr-/- Cdkn2a-/- mice), platelet activity was found to be increased (39). Copper induces cell death by targeting CDKN2A and proteins in the lipoic acid pathway (40). In this study, *CDKN2A* was significantly expressed after MI in human adipose tissue, which may indicate crosstalk among cell senescence, copper-induced cell death, and autophagy. Cyclin D2 (*CCND2*) overexpression enhances the myocardial repair functions of human iPSC-derived cardiomyocytes after MI (41, 42). As a key positive regulator of the cell cycle, inhibition of CCND2 function may induce cell cycle arrest or abnormalities in adipocytes, potentially triggering a stress response pathway distinct from canonical senescence, which could paradoxically promote the compensatory secretion of inflammatory factors like IL-6. This remains a hypothesis and will explicitly state in the text that this should be a key focus for future investigation. Chromosome 12q13.3 in *LRP1* has been correlated with spontaneous coronary artery dissection formation (43, 44). In this study, the methylation of the 82 screened overlapping genes was also investigated, demonstrating high methylation of *CDKN2A* and *CCND2* and low methylation of *DLG4* and *LRP1* after MI. Calpains mediate isoproterenol-induced hypertrophy through ubiquitin ligase *MDM2*-mediated degradation of *GRK2* (45). *IRS2* expression has been negatively correlated with apoptosis-regulatory micro RNAs and has been shown to rescue the effect of myoblast therapy after MI (46), while chronic Akt activation promotes feedback inhibition of PI3 K activity through both proteasome-dependent IRS-1 degradation and IRS-2 transcription impedance (47).

This study has some limitations. Firstly, the potential for false-negative results should be considered given the enrichment methods used. Additionally, other genes may also need to be explored. Secondly, our “hub genes” likely represent strong, common nodes in the senescence-associated secretory phenotype (SASP) or stress-response network in adipocytes within CVD contexts, and may not be exclusive to this condition. Thirdly, using 3T3-L1, a mouse pre-adipocyte cell line, to model the pathobiology of human epicardial adipose tissue indeed presents species and cell-type differences. Lastly, the sample size is small. It is hard to stratify cohorts according to different degrees of coronary artery lesions to analyze the correlation between different gene expressions and the degree of coronary artery lesions. Further research in animal models and clinical studies is required to validate these results. The observational design of this study cannot fully rule out the independent influence of these systemic metabolic factors on the gene expression profiles. A large and multicenter RCT and cohorts are still required to validate the effects of human genes on EAT senescence and MI progression. Future work is needed to dissociate these confounding effects, either through *in vitro*experiments or more stringent clinical stratification, to more purely elucidate the role of EAT senescence itself.

In conclusion, we performed bioinformatic analyses of senescence-related DEGs in human sub-epicardial adipose tissue to predict MI progression in this study. The screened hub genes, including *STAT3*, *SERPINE1*, *CDKN2A*, *DLG4*, *PTGS2*, *MDM2*, *LRP1*, *IRS2*, *PRKCD*, *CCND2*, and *CISH*, may be considered biomarkers for the prediction of and therapeutic targets for the prognosis of MI progression.

## Data Availability

Publicly available datasets were analyzed in this study. This data can be found here: https://www.ncbi.nlm.nih.gov/geo/, accession numbers: GSE18612, GSE26339, GSE35957, GSE49937 and GSE775.

## Supplementary material

The Supplementary Materialfor this article can be found online at: https://www.frontiersin.org/articles/10.3389/fcvm.2026.1759091/full#supplementary-material

Supplementary Figure S1 DEGs between EAT and SAT after CAD. (A) The volcano plot of DEGs between EAT in CAD and non-CAD subjects. (B) The volcano plot of DEGs between EAT and SAT in CAD patients. (C) The volcano plot of DEGs between EAT and SAT in non-CAD subjects. (D) The circle diagram of the GO/KEGG pathways enriched by DEGs between EAT in CAD and non-CAD subjects. (E) The circle diagram of the GO/KEGG pathways enriched by DEGs between EAT and SAT in CAD patients. (F-G) The PPI network (F) and the hub genes (G) were obtained between EAT in CAD and non-CAD subjects. (H-I) The PPI network (H) and the hub genes (I) were obtained between EAT and SAT in CAD patients.

Supplementary Figure S2 The circle diagram of the GO/KEGG pathways enriched by DEGs between EAT and SAT in non-CAD subjects.

Supplementary Figure S3 DEGs between PAT and SAT. (A) The volcano plot of DEGs between whole PAT or isolated PAT and SAT. (B) Venn diagram of whole PAT- and isolated PAT-related DEGs. (C-D) The bar plot of whole PAT-related DEGs (C) and isolated PAT-related DEGs (D). (E-F) The PPI network (E) and the hub genes (F) were obtained from whole PAT-related DEGs. (G) The immune cells abundance in whole PAT using Cibersort. (H) The upset diagram of the differentially infiltrated immune cells and whole PAT or isolated PAT DEGs. **P* < 0.05; ***P* < 0.01; ****P* < 0.001; ns, not significant.

Supplementary Figure S4 The networks of drugs-the hub DEGs was constructed.

Supplementary Figure S5 The volcano plot of the methylation of the screened 82 senescence-related genes in EAT.

Supplementary Figure S6 The joint ROC of 11 validated hub genes about patients with CAD and severe CAD.

Supplementary Figure S7 The knockdown efficiency of hub genes shRNA. *N* = 3 each group.
