# Supplementary figures and images for: Senescence-related epicardial adipocyte genes lead to immune infiltration and myocardial infarction progression
Source: Front Cardiovasc Med. 2026 Mar 5;13:1759091. doi: 10.3389/fcvm.2026.1759091 (PMC12999425; doi:10.3389/fcvm.2026.1759091)

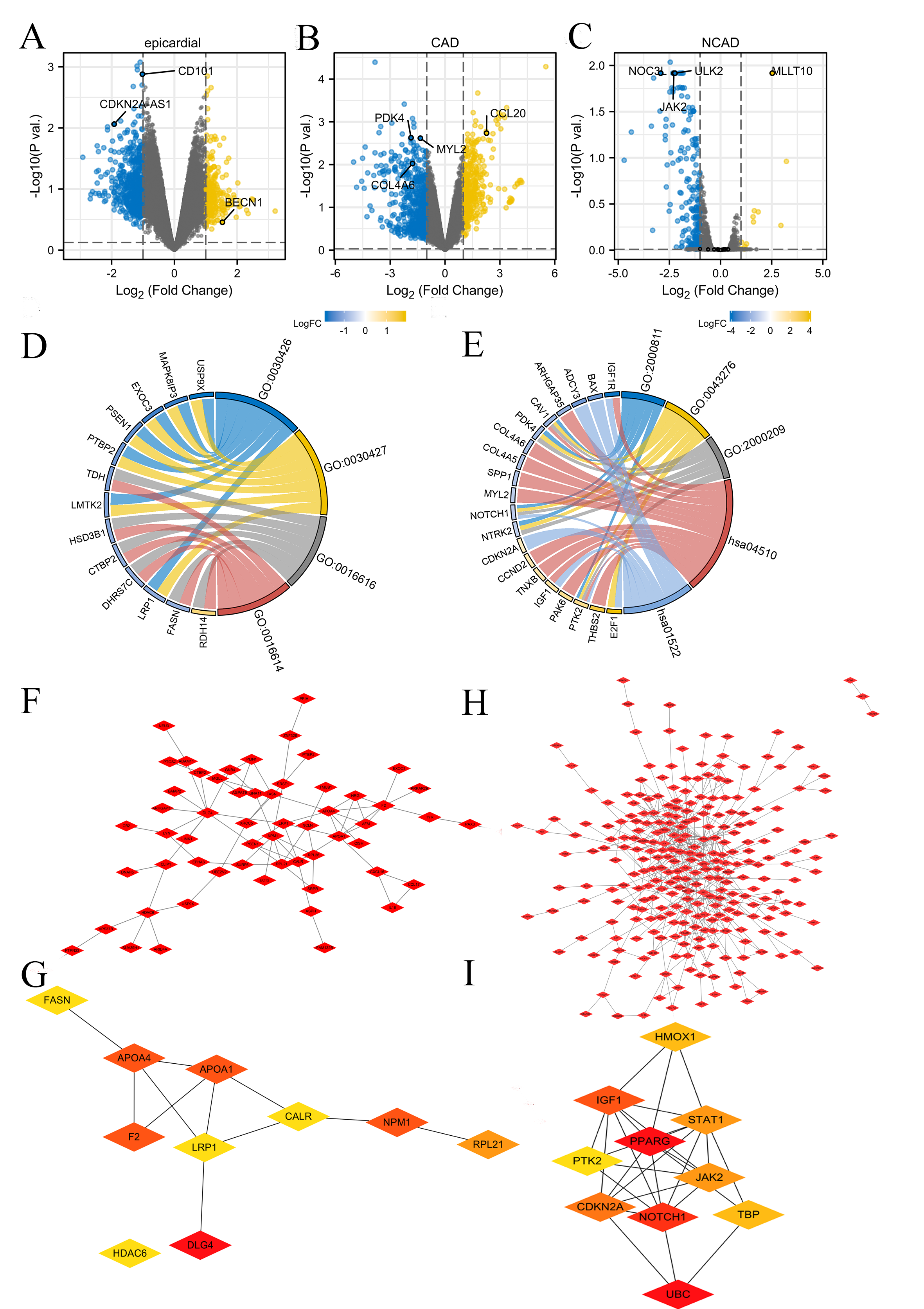

Supplement: Supplementary Figure S1 — DEGs between EAT and SAT after CAD. (A) The volcano plot of DEGs between EAT in CAD and non-CAD subjects. (B) The volcano plot of DEGs between EAT and SAT in CAD patients. (C) The volcano plot of DEGs between EAT and SAT in non-CAD subjects. (D) The circle diagram of the GO/KEGG pathways enriched by DEGs between EAT in CAD and non-CAD subjects. (E) The circle diagram of the GO/KEGG pathways enriched by DEGs between EAT and SAT in CAD patients. (F-G) The PPI network (F) and the hub genes (G) were obtained between EAT in CAD and non-CAD subjects. (H-I) The PPI network (H) and the hub genes (I) were obtained between EAT and SAT in CAD patients. [file Image1.tif]

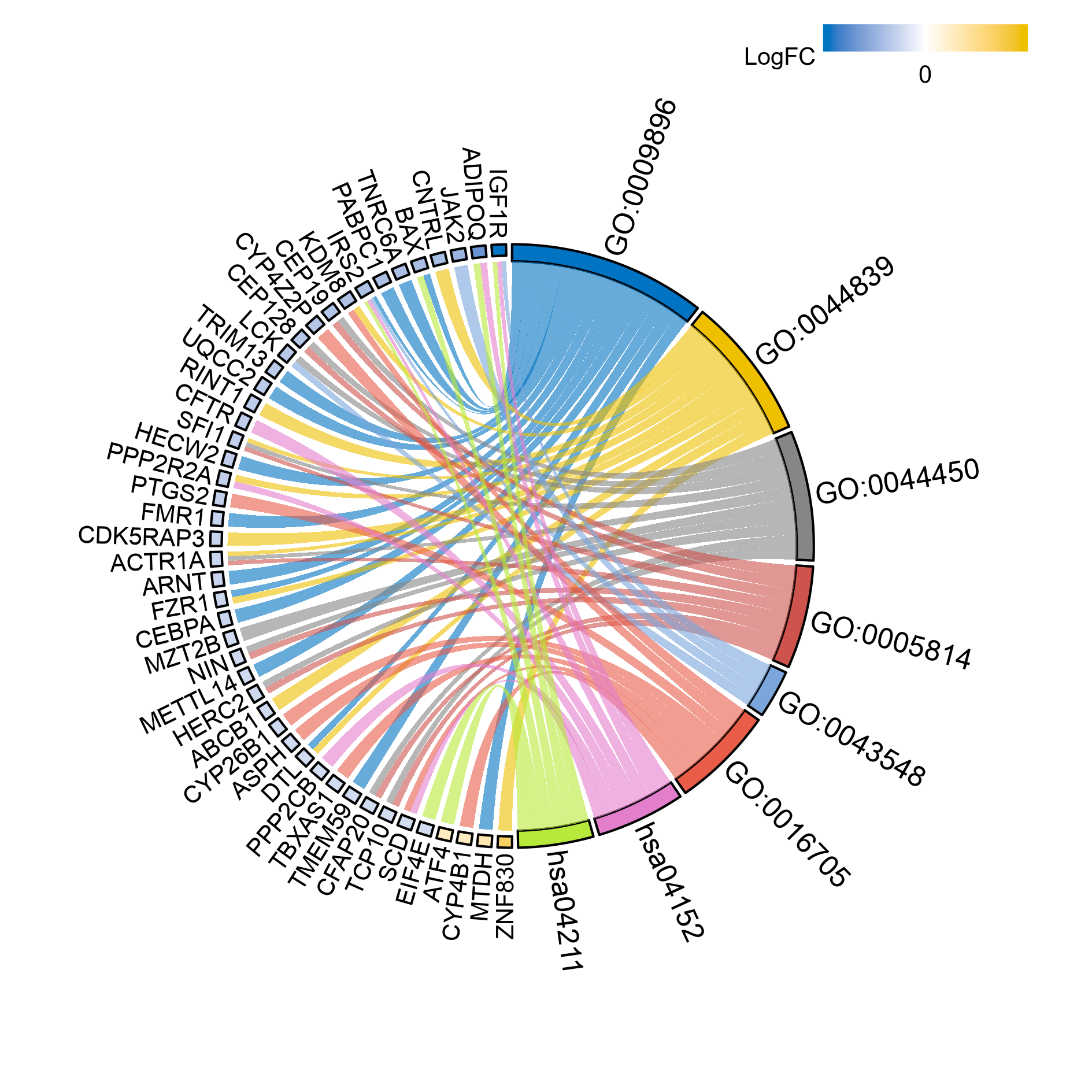

Supplement: Supplementary Figure S2 — The circle diagram of the GO/KEGG pathways enriched by DEGs between EAT and SAT in non-CAD subjects. [file Image2.tiff]

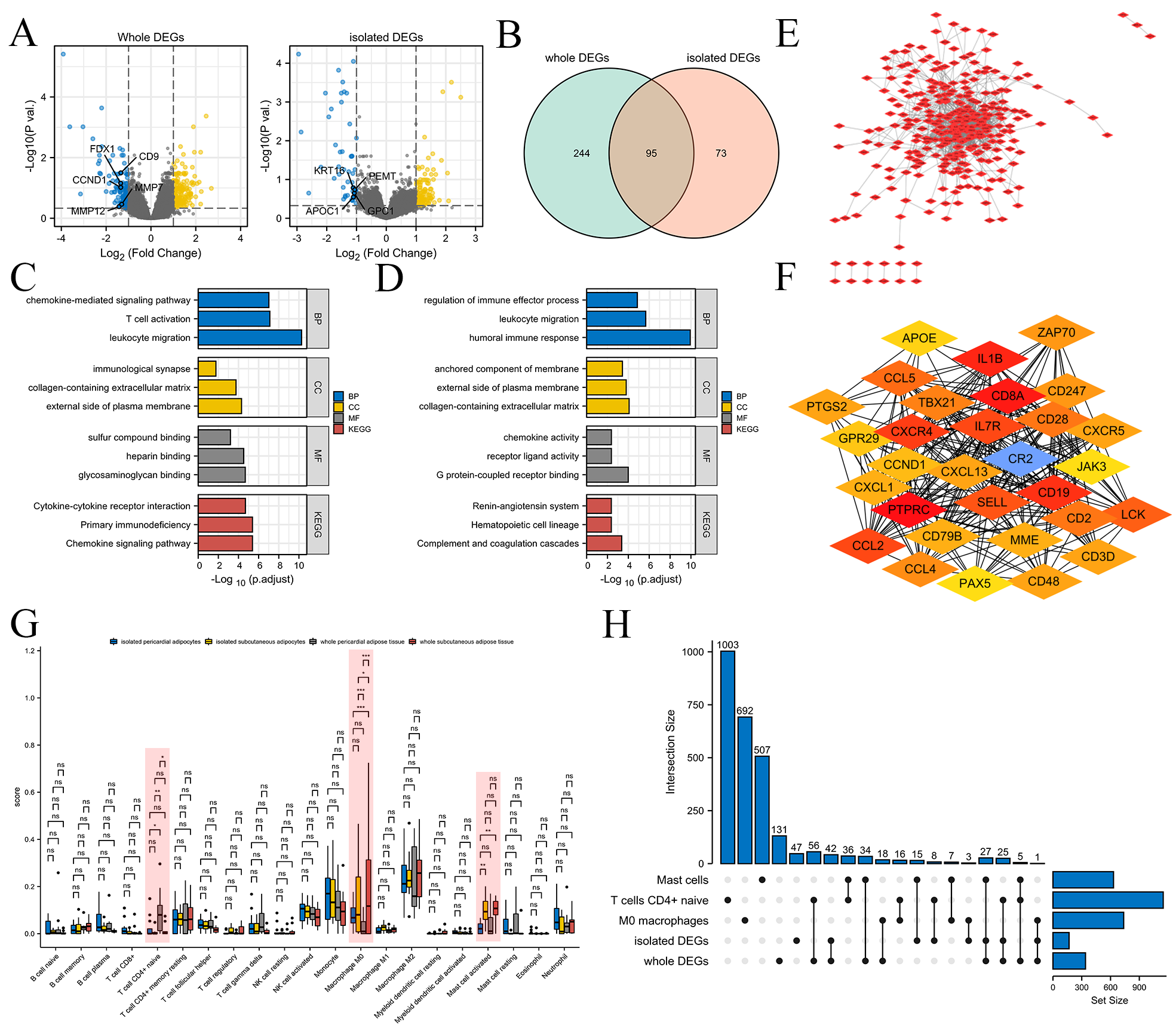

Supplement: Supplementary Figure S3 — DEGs between PAT and SAT. (A) The volcano plot of DEGs between whole PAT or isolated PAT and SAT. (B) Venn diagram of whole PAT- and isolated PAT-related DEGs. (C-D) The bar plot of whole PAT-related DEGs (C) and isolated PAT-related DEGs (D). (E-F) The PPI network (E) and the hub genes (F) were obtained from whole PAT-related DEGs. (G) The immune cells abundance in whole PAT using Cibersort. (H) The upset diagram of the differentially infiltrated immune cells and whole PAT or isolated PAT DEGs. *P < 0.05; **P < 0.01; ***P < 0.001; ns, not significant. [file Image3.tif]

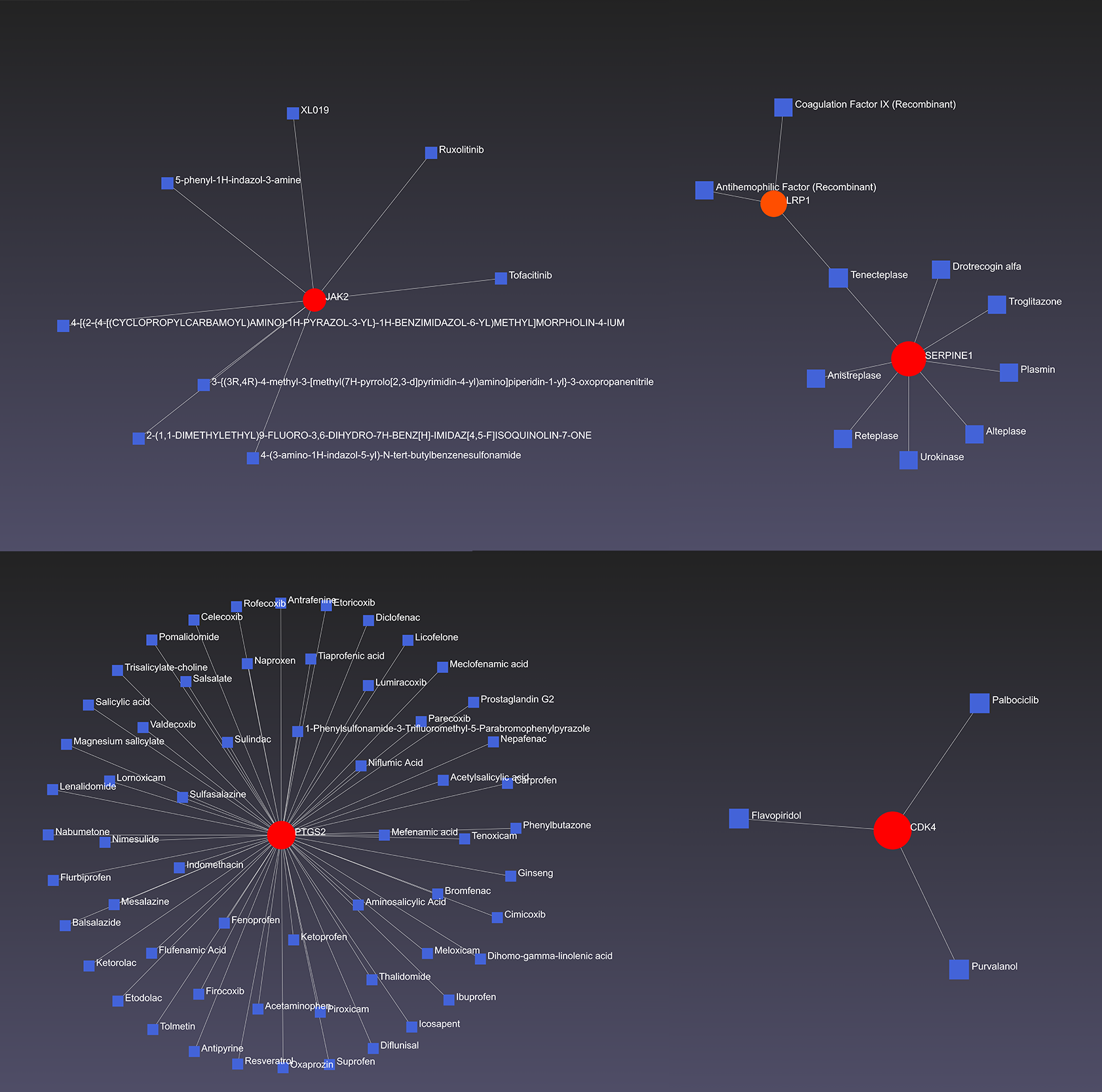

Supplement: Supplementary Figure S4 — The networks of drugs-the hub DEGs was constructed. [file Image4.tif]

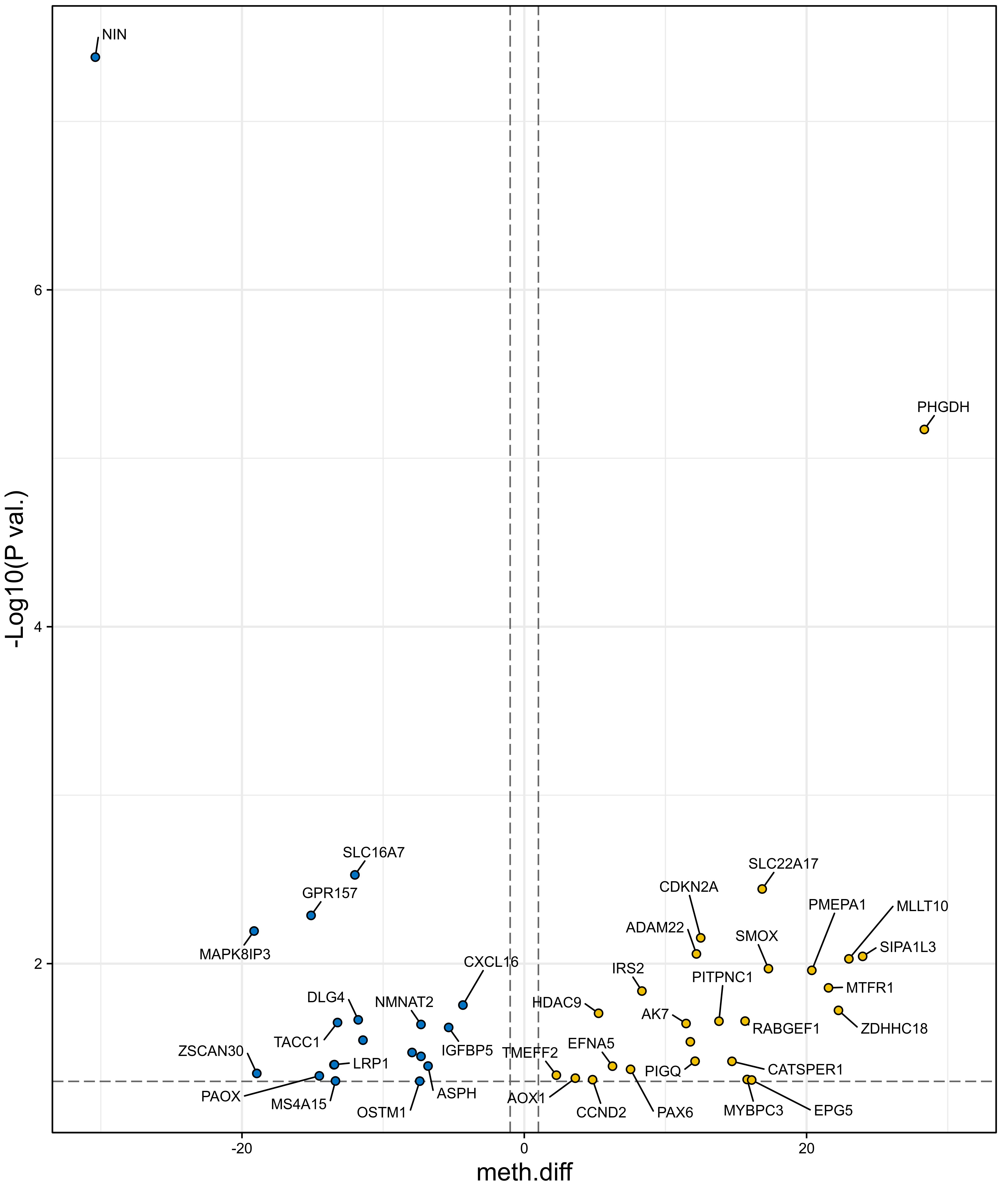

Supplement: Supplementary Figure S5 — The volcano plot of the methylation of the screened 82 senescence-related genes in EAT. [file Image5.tiff]

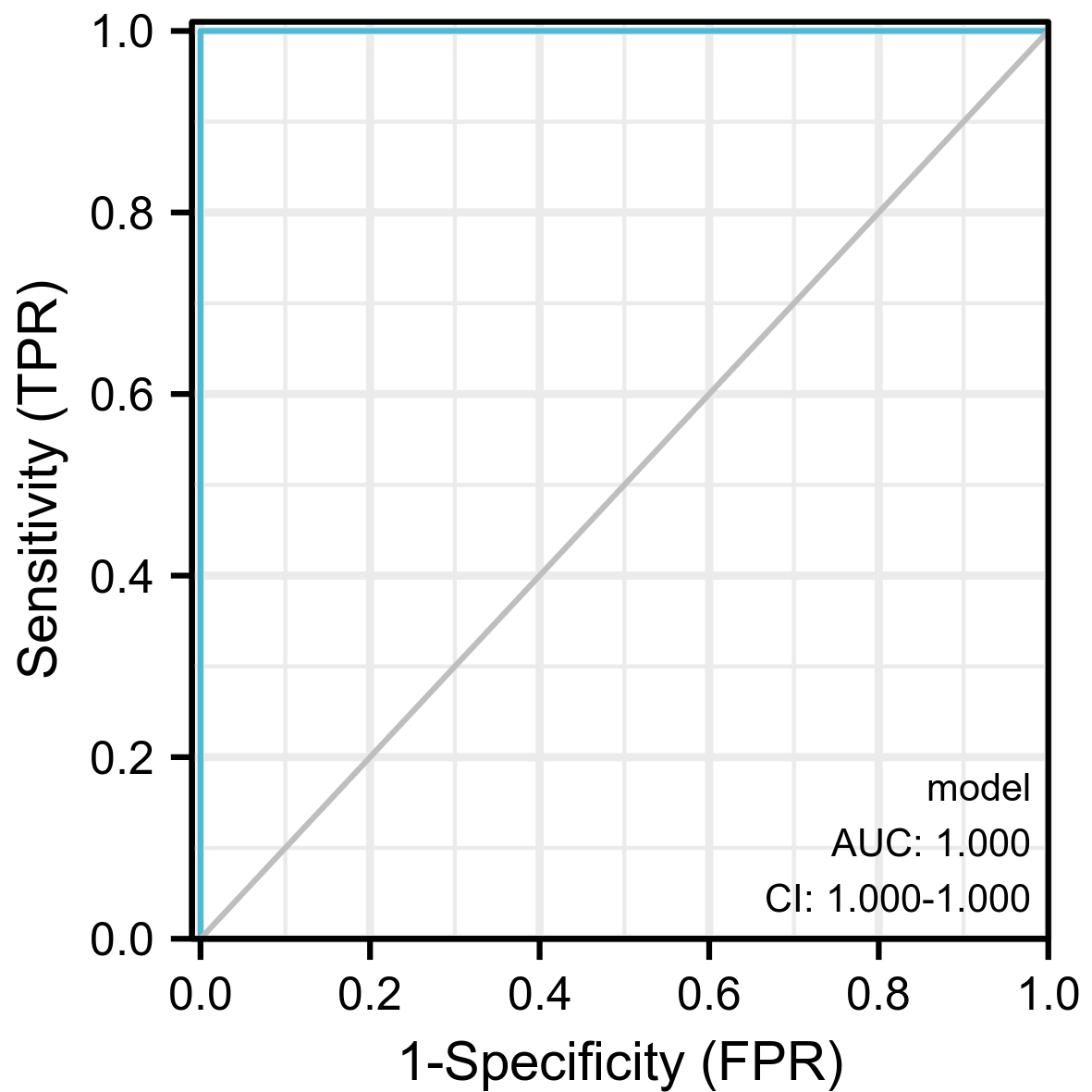

Supplement: Supplementary Figure S6 — The joint ROC of 11 validated hub genes about patients with CAD and severe CAD. [file Image6.tiff]

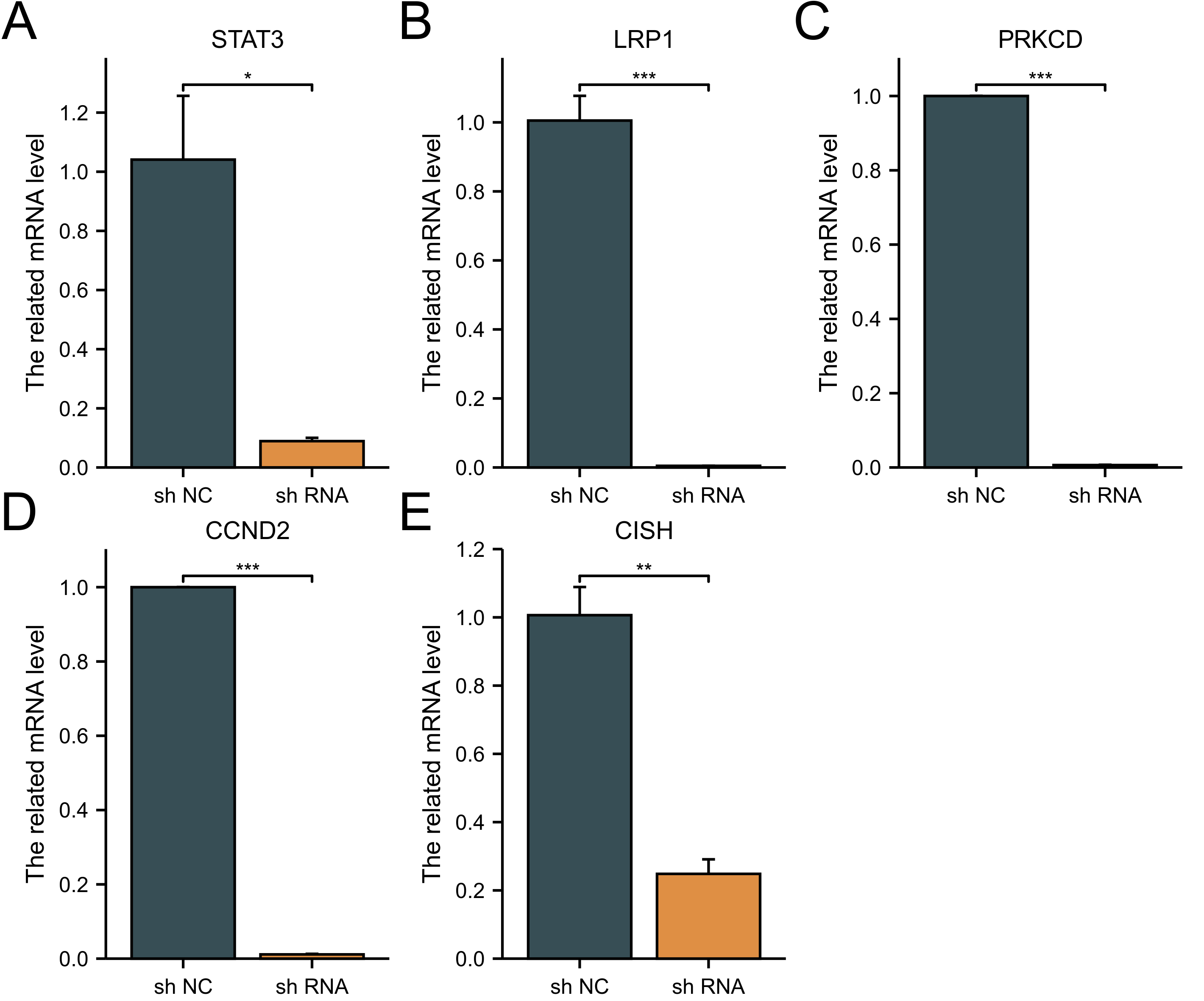

Supplement: Supplementary Figure S7 — The knockdown efficiency of hub genes shRNA. N = 3 each group. [file Image7.tif]
