# Supplementary material for: Senescence-related epicardial adipocyte genes lead to immune infiltration and myocardial infarction progression
Source: Front Cardiovasc Med. 2026 Mar 5;13:1759091. doi: 10.3389/fcvm.2026.1759091 (PMC12999425; doi:10.3389/fcvm.2026.1759091)
Supplement: Supplementary file 8 [file Table1.docx]

Supplementary Table 1. Specific human primers used for quantitative real-time PCR.

| Genes | Forward (5’-3’) | Reverse (5’-3’) |
| --- | --- | --- |
| STAT3 | CAGCAGCTTGACACACGGTA | AAACACCAAAGTGGCATGTGA |
| JAK2 | ATCCACCCAACCATGTCTTCC | ATTCCATGCCGATAGGCTCTG |
| SERPINE1 | ATTTGACGACGAATCGGACCC | GTTCTTGCGGTCTTTCTGGGA |
| CDKN2A | CTTCCTCGGGTGCCGATAC | ACCCCTTCATTGCTACTCGAT |
| DLG4 | TCGGTGACGACCCATCCAT | GCACGTCCACTTCATTTACAAAC |
| APOE | GTTGCTGGTCACATTCCTGG | GCAGGTAATCCCAAAAGCGAC |
| PTGS2 | TAAGTGCGATTGTACCCGGAC | TTTGTAGCCATAGTCAGCATTGT |
| SPP1 | CTCCATTGACTCGAACGACTC | CAGGTCTGCGAAACTTCTTAGAT |
| MDM2 | GGCAGGGGAGAGTGATACAGA | GAAGCCAATTCTCACGAAGGG |
| LRP1 | AACGAGCATAACTGCCTGGG | CGTACACTGAGCACTCATCAAA |
| IRS2 | CACCTCCCCACGACAGTTG | TCGATGGCGATGTAGTTGAGA |
| PRKCD | AACGCTGCCATCCACAAGAAA | TTTAATCCCTGCTTCACCAGTC |
| CCND2 | CTGTCTCTGATCCGCAAGCAT | GGTGGGTACATGGCAAACTTAAA |
| CDK4 | ATGGCTACCTCTCGATATGAGC | CATTGGGGACTCTCACACTCT |
| CISH | TGCCAGAAGGCACGTTCTTAG | GCCACGAGTGGTTTTCACTG |
| B-actin | CATGTACGTTGCTATCCAGGC | CTCCTTAATGTCACGCACGAT |
