# Supplementary material for: Senescence-related epicardial adipocyte genes lead to immune infiltration and myocardial infarction progression
Source: Front Cardiovasc Med. 2026 Mar 5;13:1759091. doi: 10.3389/fcvm.2026.1759091 (PMC12999425; doi:10.3389/fcvm.2026.1759091)
Supplement: Supplementary file 9 [file Table2.docx]

Supplementary Table 2. The GO/KEGG pathways enriched by DEGs between EAT in CAD and non-CAD patients.

| ONTOLOGY | ID | Description | GeneRatio | BgRatio | pvalue | p.adjust | qvalue | geneID | Count | zscore |
| --- | --- | --- | --- | --- | --- | --- | --- | --- | --- | --- |
| CC | GO:0030426 | growth cone | 7/135 | 171/19717 | 0.000178074 | 0.03571245 | 0.030991067 | LRP1/LMTK2/PTBP2/PSEN1/EXOC3/MAPK8IP3/USP9X | 7 | -2.645751311 |
| CC | GO:0030427 | site of polarized growth | 7/135 | 176/19717 | 0.000212574 | 0.03571245 | 0.030991067 | LRP1/LMTK2/PTBP2/PSEN1/EXOC3/MAPK8IP3/USP9X | 7 | -2.645751311 |
| MF | GO:0016616 | oxidoreductase activity, acting on the CH-OH group of donors, NAD or NADP as acceptor | 6/132 | 119/17697 | 0.000267689 | 0.083792102 | 0.0827808 | RDH14/FASN/DHRS7C/CTBP2/HSD3B1/TDH | 6 | -1.632993162 |
| MF | GO:0016614 | oxidoreductase activity, acting on CH-OH group of donors | 6/132 | 128/17697 | 0.00039618 | 0.083792102 | 0.0827808 | RDH14/FASN/DHRS7C/CTBP2/HSD3B1/TDH | 6 | -1.632993162 |

DEGs, Different Expressed Genes; EAT, epicardial adipose tissue; CAD, coronary artery disease; GO, Gene ONTOLOGY; BP, Biological Process; CC, cellular component; MF, Molecular Function; KEGG, Kyoto Encyclopedia of Genes and Genomes.
