# Supplementary material for: Senescence-related epicardial adipocyte genes lead to immune infiltration and myocardial infarction progression
Source: Front Cardiovasc Med. 2026 Mar 5;13:1759091. doi: 10.3389/fcvm.2026.1759091 (PMC12999425; doi:10.3389/fcvm.2026.1759091)
Supplement: Supplementary file 10 [file Table3.docx]

Supplementary Table 3. The GO/KEGG pathways enriched by DEGs between EAT and SAT in CAD patients.

| ONTOLOGY | ID | Description | GeneRatio | BgRatio | pvalue | p.adjust | qvalue | geneID | Count | zscore |
| --- | --- | --- | --- | --- | --- | --- | --- | --- | --- | --- |
| BP | GO:2000811 | negative regulation of anoikis | 5/330 | 17/18670 | 8.69753E-06 | 0.033720326 | 0.030990674 | PDK4/PTK2/CAV1/NOTCH1/NTRK2 | 5 | -1.341640786 |
| BP | GO:0043276 | anoikis | 6/330 | 34/18670 | 2.58102E-05 | 0.050033106 | 0.045982939 | PDK4/PTK2/CAV1/NOTCH1/E2F1/NTRK2 | 6 | -0.816496581 |
| BP | GO:2000209 | regulation of anoikis | 5/330 | 24/18670 | 5.39633E-05 | 0.069738567 | 0.064093249 | PDK4/PTK2/CAV1/NOTCH1/NTRK2 | 5 | -1.341640786 |
| KEGG | hsa04510 | Focal adhesion | 13/157 | 201/8076 | 0.000139147 | 0.035900027 | 0.033688316 | THBS2/MYL2/PTK2/TNXB/CAV1/SPP1/COL4A6/PAK6/IGF1/IGF1R/ARHGAP35/CCND2/COL4A5 | 13 | -0.277350098 |
| KEGG | hsa01522 | Endocrine resistance | 8/157 | 98/8076 | 0.000610641 | 0.078772664 | 0.073919677 | BAX/PTK2/ADCY3/NOTCH1/CDKN2A/E2F1/IGF1/IGF1R | 8 | 0 |

DEGs, Different Expressed Genes; EAT, epicardial adipose tissue; SAT, subcutaneous adipose tissue; CAD, coronary artery disease; GO, Gene ONTOLOGY; BP, Biological Process; CC, cellular component; MF, Molecular Function; KEGG, Kyoto Encyclopedia of Genes and Genomes.
