# Supplementary material for: Senescence-related epicardial adipocyte genes lead to immune infiltration and myocardial infarction progression
Source: Front Cardiovasc Med. 2026 Mar 5;13:1759091. doi: 10.3389/fcvm.2026.1759091 (PMC12999425; doi:10.3389/fcvm.2026.1759091)
Supplement: Supplementary file 11 [file Table4.docx]

Supplementary Table 4. The GO/KEGG pathways enriched by DEGs between EAT and SAT in non-CAD subjects.

| ONTOLOGY | ID | Description | GeneRatio | BgRatio | pvalue | p.adjust | qvalue | geneID | Count | zscore |
| --- | --- | --- | --- | --- | --- | --- | --- | --- | --- | --- |
| BP | GO:0009896 | positive regulation of catabolic process | 15/202 | 423/18670 | 6.00473E-05 | 0.088357714 | 0.079240656 | PABPC1/UQCC2/BAX/FZR1/METTL14/FMR1/TMEM59/DTL/CEBPA/TNRC6A/MTDH/IRS2/ARNT/HECW2/TRIM13 | 15 | -3.356585567 |
| BP | GO:0044839 | cell cycle G2/M phase transition | 11/202 | 266/18670 | 0.000157804 | 0.088357714 | 0.079240656 | CNTRL/KDM8/ZNF830/FZR1/CDK5RAP3/DTL/PPP2R2A/RINT1/ACTR1A/ABCB1/SFI1 | 11 | -2.713602101 |
| BP | GO:0000077 | DNA damage checkpoint | 8/202 | 145/18670 | 0.000183663 | 0.088357714 | 0.079240656 | E2F4/BAX/ZNF830/FZR1/CDK5RAP3/DTL/EME1/RINT1 | 8 | -2.121320344 |
| BP | GO:0019371 | cyclooxygenase pathway | 3/202 | 11/18670 | 0.000193133 | 0.088357714 | 0.079240656 | PTGES3/TBXAS1/PTGS2 | 3 | -1.732050808 |
| BP | GO:0070102 | interleukin-6-mediated signaling pathway | 4/202 | 29/18670 | 0.000255564 | 0.088357714 | 0.079240656 | JAK2/STAT1/SMAD4/CEBPA | 4 | -2 |
| BP | GO:0048871 | multicellular organismal homeostasis | 15/202 | 485/18670 | 0.000268666 | 0.088357714 | 0.079240656 | JAK2/ADIPOQ/SIRT6/ADD1/BAX/IGF1R/ZNF830/PRCP/PTH/SCD/B2M/PTGS2/ATF4/CYP26B1/CFTR | 15 | -2.840187787 |
| BP | GO:0042593 | glucose homeostasis | 10/202 | 241/18670 | 0.000305173 | 0.088357714 | 0.079240656 | ADIPOQ/SIRT6/IGF1R/NGFR/PRCP/NUCKS1/IGFBP5/CEBPA/IRS2/CFTR | 10 | -3.16227766 |
| BP | GO:0033500 | carbohydrate homeostasis | 10/202 | 242/18670 | 0.000315381 | 0.088357714 | 0.079240656 | ADIPOQ/SIRT6/IGF1R/NGFR/PRCP/NUCKS1/IGFBP5/CEBPA/IRS2/CFTR | 10 | -3.16227766 |
| BP | GO:0031570 | DNA integrity checkpoint | 8/202 | 157/18670 | 0.000315577 | 0.088357714 | 0.079240656 | E2F4/BAX/ZNF830/FZR1/CDK5RAP3/DTL/EME1/RINT1 | 8 | -2.121320344 |
| BP | GO:0043271 | negative regulation of ion transport | 8/202 | 157/18670 | 0.000315577 | 0.088357714 | 0.079240656 | PKD2/FMR1/PTH/SNX16/IRS2/PTGS2/ATF4/HECW2 | 8 | -2.121320344 |
| BP | GO:0031572 | G2 DNA damage checkpoint | 4/202 | 31/18670 | 0.000332893 | 0.088357714 | 0.079240656 | FZR1/CDK5RAP3/DTL/RINT1 | 4 | -2 |
| BP | GO:0000086 | G2/M transition of mitotic cell cycle | 10/202 | 247/18670 | 0.000370801 | 0.088357714 | 0.079240656 | CNTRL/KDM8/ZNF830/CDK5RAP3/DTL/PPP2R2A/RINT1/ACTR1A/ABCB1/SFI1 | 10 | -2.529822128 |
| BP | GO:0034764 | positive regulation of transmembrane transport | 9/202 | 204/18670 | 0.000390657 | 0.088357714 | 0.079240656 | ADIPOQ/PKD2/BAX/PTH/CACNG3/IRS2/ABCB1/LRRC55/CFTR | 9 | -3 |
| BP | GO:0030837 | negative regulation of actin filament polymerization | 5/202 | 58/18670 | 0.000405954 | 0.088357714 | 0.079240656 | ADD1/CAPZA1/SPTBN5/LMOD3/FHOD3 | 5 | -2.236067977 |
| BP | GO:0072425 | signal transduction involved in G2 DNA damage checkpoint | 3/202 | 14/18670 | 0.00041597 | 0.088357714 | 0.079240656 | FZR1/DTL/RINT1 | 3 | -1.732050808 |
| BP | GO:1903902 | positive regulation of viral life cycle | 5/202 | 61/18670 | 0.000513445 | 0.088357714 | 0.079240656 | PABPC1/NUCKS1/FMR1/CAV2/CHMP3 | 5 | -2.236067977 |
| BP | GO:0051770 | positive regulation of nitric-oxide synthase biosynthetic process | 3/202 | 15/18670 | 0.000515828 | 0.088357714 | 0.079240656 | JAK2/STAT1/AKAP12 | 3 | -0.577350269 |
| BP | GO:0072075 | metanephric mesenchyme development | 3/202 | 15/18670 | 0.000515828 | 0.088357714 | 0.079240656 | PKD2/STAT1/SMAD4 | 3 | -1.732050808 |
| BP | GO:0060537 | muscle tissue development | 13/202 | 408/18670 | 0.000521236 | 0.088357714 | 0.079240656 | SIRT6/MYOM1/UQCC2/HDAC9/PKD2/LMOD3/ADAMTS9/SMAD4/IGFBP5/CAV2/CYP26B1/FHOD3/FOXP2 | 13 | -3.605551275 |
| BP | GO:0010830 | regulation of myotube differentiation | 5/202 | 62/18670 | 0.000553633 | 0.088357714 | 0.079240656 | HDAC9/LMOD3/PLPP7/CAPN2/CYP26B1 | 5 | -2.236067977 |
| BP | GO:0031331 | positive regulation of cellular catabolic process | 12/202 | 361/18670 | 0.000588354 | 0.088357714 | 0.079240656 | PABPC1/UQCC2/BAX/METTL14/FMR1/TMEM59/CEBPA/TNRC6A/MTDH/IRS2/ARNT/TRIM13 | 12 | -2.886751346 |
| BP | GO:0000075 | cell cycle checkpoint | 9/202 | 216/18670 | 0.000590842 | 0.088357714 | 0.079240656 | E2F4/BAX/ZNF830/FZR1/CDK5RAP3/DTL/EME1/RINT1/BCL2L1 | 9 | -1.666666667 |
| BP | GO:0044773 | mitotic DNA damage checkpoint | 6/202 | 97/18670 | 0.000650144 | 0.092998842 | 0.083402897 | E2F4/BAX/ZNF830/CDK5RAP3/EME1/RINT1 | 6 | -1.632993162 |
| CC | GO:0044450 | microtubule organizing center part | 10/209 | 185/19717 | 2.91713E-05 | 0.011610172 | 0.01044025 | CEP19/MZT2B/NIN/CEP128/HERC2/ACTR1A/LCK/TCP10/SFI1/CFAP20 | 10 | -3.16227766 |
| CC | GO:0005814 | centriole | 8/209 | 139/19717 | 0.000119726 | 0.023825509 | 0.021424685 | CEP19/NIN/CEP128/HERC2/ACTR1A/TCP10/SFI1/CFAP20 | 8 | -2.828427125 |
| CC | GO:0030658 | transport vesicle membrane | 9/209 | 208/19717 | 0.000389818 | 0.051715877 | 0.046504624 | RAB26/SEMA4C/CLTB/TMED7/HLA-DPB1/B2M/SPRED2/SYNGR4/BCL2L1 | 9 | -1.666666667 |
| CC | GO:0044448 | cell cortex part | 8/209 | 184/19717 | 0.000792605 | 0.078864159 | 0.070917255 | CAPZA1/SPTBN5/PKD2/CLTB/CAPN2/EXOC3/ACTR1A/ASPH | 8 | -2.828427125 |
| CC | GO:0044853 | plasma membrane raft | 6/209 | 109/19717 | 0.00108052 | 0.086009355 | 0.077342451 | JAK2/ADD1/CAV2/PRTN3/MAL/PTGS2 | 6 | -2.449489743 |
| CC | GO:0034708 | methyltransferase complex | 6/209 | 113/19717 | 0.001301932 | 0.086361508 | 0.077659119 | HDAC9/METTL14/MAX/TEX10/CLNS1A/SNRPF | 6 | -1.632993162 |
| CC | GO:0005938 | cell cortex | 10/209 | 308/19717 | 0.001717684 | 0.091631499 | 0.082398069 | CAPZA1/SPTBN5/PKD2/CLTB/FRYL/CAPN2/EXOC3/ACTR1A/ASPH/AKAP12 | 10 | -2.529822128 |
| CC | GO:0045121 | membrane raft | 10/209 | 315/19717 | 0.002024792 | 0.091631499 | 0.082398069 | JAK2/ADD1/CAPN2/SDCBP/CAV2/PRTN3/MAL/LCK/PTGS2/BTK | 10 | -3.16227766 |
| CC | GO:0098857 | membrane microdomain | 10/209 | 316/19717 | 0.002072069 | 0.091631499 | 0.082398069 | JAK2/ADD1/CAPN2/SDCBP/CAV2/PRTN3/MAL/LCK/PTGS2/BTK | 10 | -3.16227766 |
| MF | GO:0043548 | phosphatidylinositol 3-kinase binding | 4/197 | 30/17697 | 0.000325366 | 0.098446465 | 0.09513734 | JAK2/IGF1R/IRS2/LCK | 4 | -2 |
| MF | GO:0016705 | oxidoreductase activity, acting on paired donors, with incorporation or reduction of molecular oxygen | 8/197 | 159/17697 | 0.000413641 | 0.098446465 | 0.09513734 | KDM8/SCD/TBXAS1/CYP4Z2P/ASPH/PTGS2/CYP26B1/CYP4B1 | 8 | -2.121320344 |
| KEGG | hsa04152 | AMPK signaling pathway | 7/91 | 120/8076 | 0.000387169 | 0.051145728 | 0.046575791 | ADIPOQ/IGF1R/PPP2R2A/SCD/PPP2CB/IRS2/CFTR | 7 | -2.645751311 |
| KEGG | hsa04211 | Longevity regulating pathway | 6/91 | 89/8076 | 0.000475774 | 0.051145728 | 0.046575791 | ADIPOQ/BAX/IGF1R/IRS2/EIF4E/ATF4 | 6 | -1.632993162 |

DEGs, Different Expressed Genes; EAT, epicardial adipose tissue; SAT, subcutaneous adipose tissue; CAD, coronary artery disease; GO, Gene ONTOLOGY; BP, Biological Process; CC, cellular component; MF, Molecular Function; KEGG, Kyoto Encyclopedia of Genes and Genomes.
