# Supplementary material for: Senescence-related epicardial adipocyte genes lead to immune infiltration and myocardial infarction progression
Source: Front Cardiovasc Med. 2026 Mar 5;13:1759091. doi: 10.3389/fcvm.2026.1759091 (PMC12999425; doi:10.3389/fcvm.2026.1759091)
Supplement: Supplementary file 12 [file Table5.docx]

Supplementary Table 5. The GO/KEGG pathways enriched by DEGs of whole PAT compared to SAT.

| ONTOLOGY | ID | Description | GeneRatio | BgRatio | pvalue | p.adjust | qvalue |
| --- | --- | --- | --- | --- | --- | --- | --- |
| BP | GO:0050900 | leukocyte migration | 36/289 | 499/18670 | 1.44e-14 | 5.43e-11 | 4.04e-11 |
| BP | GO:0042110 | T cell activation | 30/289 | 464/18670 | 4.07e-11 | 7.70e-08 | 5.72e-08 |
| BP | GO:0070098 | chemokine-mediated signaling pathway | 14/289 | 88/18670 | 7.64e-11 | 9.63e-08 | 7.16e-08 |
| BP | GO:1990868 | response to chemokine | 14/289 | 97/18670 | 2.93e-10 | 2.13e-07 | 1.58e-07 |
| BP | GO:1990869 | cellular response to chemokine | 14/289 | 97/18670 | 2.93e-10 | 2.13e-07 | 1.58e-07 |
| CC | GO:0009897 | external side of plasma membrane | 22/296 | 393/19717 | 1.38e-07 | 5.00e-05 | 4.24e-05 |
| CC | GO:0062023 | collagen-containing extracellular matrix | 21/296 | 406/19717 | 9.70e-07 | 1.76e-04 | 1.49e-04 |
| CC | GO:0001772 | immunological synapse | 5/296 | 36/19717 | 1.90e-04 | 0.017 | 0.015 |
| CC | GO:0070820 | tertiary granule | 10/296 | 164/19717 | 1.92e-04 | 0.017 | 0.015 |
| CC | GO:0098589 | membrane region | 14/296 | 328/19717 | 4.68e-04 | 0.034 | 0.029 |
| MF | GO:0005539 | glycosaminoglycan binding | 18/287 | 229/17697 | 3.87e-08 | 2.13e-05 | 1.86e-05 |
| MF | GO:0008201 | heparin binding | 15/287 | 169/17697 | 1.11e-07 | 3.04e-05 | 2.65e-05 |
| MF | GO:1901681 | sulfur compound binding | 16/287 | 250/17697 | 3.43e-06 | 6.28e-04 | 5.48e-04 |
| MF | GO:0008009 | chemokine activity | 7/287 | 49/17697 | 1.32e-05 | 0.002 | 0.001 |
| MF | GO:0005125 | cytokine activity | 14/287 | 220/17697 | 1.50e-05 | 0.002 | 0.001 |
| KEGG | hsa04062 | Chemokine signaling pathway | 18/158 | 192/8076 | 3.11e-08 | 4.09e-06 | 3.64e-06 |
| KEGG | hsa05340 | Primary immunodeficiency | 9/158 | 38/8076 | 3.35e-08 | 4.09e-06 | 3.64e-06 |
| KEGG | hsa04060 | Cytokine-cytokine receptor interaction | 21/158 | 295/8076 | 2.51e-07 | 2.04e-05 | 1.82e-05 |
| KEGG | hsa04640 | Hematopoietic cell lineage | 12/158 | 99/8076 | 4.41e-07 | 2.40e-05 | 2.13e-05 |
| KEGG | hsa04061 | Viral protein interaction with cytokine and cytokine receptor | 12/158 | 100/8076 | 4.92e-07 | 2.40e-05 | 2.13e-05 |

DEGs, Different Expressed Genes; PAT, pericardial adipose tissue; SAT, subcutaneous adipose tissue; CAD, coronary artery disease; GO, Gene ONTOLOGY; BP, Biological Process; CC, cellular component; MF, Molecular Function; KEGG, Kyoto Encyclopedia of Genes and Genomes.
