# Supplementary material for: Senescence-related epicardial adipocyte genes lead to immune infiltration and myocardial infarction progression
Source: Front Cardiovasc Med. 2026 Mar 5;13:1759091. doi: 10.3389/fcvm.2026.1759091 (PMC12999425; doi:10.3389/fcvm.2026.1759091)
Supplement: Supplementary file 13 [file Table6.docx]

Supplementary Table 6. The GO/KEGG pathways enriched by DEGs of isolated PAT compared to SAT.

| ONTOLOGY | ID | Description | GeneRatio | BgRatio | pvalue | p.adjust | qvalue |
| --- | --- | --- | --- | --- | --- | --- | --- |
| BP | GO:0006959 | humoral immune response | 22/143 | 356/18670 | 4.00e-14 | 1.17e-10 | 9.01e-11 |
| BP | GO:0050900 | leukocyte migration | 20/143 | 499/18670 | 1.48e-09 | 2.16e-06 | 1.66e-06 |
| BP | GO:0002697 | regulation of immune effector process | 18/143 | 458/18670 | 1.42e-08 | 1.38e-05 | 1.06e-05 |
| BP | GO:0051249 | regulation of lymphocyte activation | 18/143 | 485/18670 | 3.40e-08 | 2.16e-05 | 1.66e-05 |
| BP | GO:0002696 | positive regulation of leukocyte activation | 16/143 | 380/18670 | 3.69e-08 | 2.16e-05 | 1.66e-05 |
| CC | GO:0062023 | collagen-containing extracellular matrix | 15/148 | 406/19717 | 4.27e-07 | 8.45e-05 | 7.95e-05 |
| CC | GO:0009897 | external side of plasma membrane | 14/148 | 393/19717 | 1.62e-06 | 1.61e-04 | 1.51e-04 |
| CC | GO:0031225 | anchored component of membrane | 9/148 | 170/19717 | 5.60e-06 | 3.70e-04 | 3.48e-04 |
| CC | GO:0045121 | membrane raft | 11/148 | 315/19717 | 2.66e-05 | 0.001 | 0.001 |
| CC | GO:0098857 | membrane microdomain | 11/148 | 316/19717 | 2.74e-05 | 0.001 | 0.001 |
| MF | GO:0001664 | G protein-coupled receptor binding | 13/139 | 280/17697 | 3.25e-07 | 1.03e-04 | 8.99e-05 |
| MF | GO:0048018 | receptor ligand activity | 14/139 | 482/17697 | 2.68e-05 | 0.004 | 0.004 |
| MF | GO:0008009 | chemokine activity | 5/139 | 49/17697 | 4.02e-05 | 0.004 | 0.004 |
| MF | GO:0005125 | cytokine activity | 9/139 | 220/17697 | 6.10e-05 | 0.005 | 0.004 |
| MF | GO:0042379 | chemokine receptor binding | 5/139 | 66/17697 | 1.69e-04 | 0.009 | 0.008 |
| KEGG | hsa04610 | Complement and coagulation cascades | 8/83 | 85/8076 | 2.24e-06 | 4.32e-04 | 3.99e-04 |
| KEGG | hsa04640 | Hematopoietic cell lineage | 7/83 | 99/8076 | 6.52e-05 | 0.004 | 0.004 |
| KEGG | hsa04614 | Renin-angiotensin system | 4/83 | 23/8076 | 7.92e-05 | 0.004 | 0.004 |
| KEGG | hsa04064 | NF-kappa B signaling pathway | 7/83 | 104/8076 | 8.92e-05 | 0.004 | 0.004 |
| KEGG | hsa04060 | Cytokine-cytokine receptor interaction | 11/83 | 295/8076 | 1.98e-04 | 0.008 | 0.007 |

DEGs, Different Expressed Genes; PAT, pericardial adipose tissue; SAT, subcutaneous adipose tissue; CAD, coronary artery disease; GO, Gene ONTOLOGY; BP, Biological Process; CC, cellular component; MF, Molecular Function; KEGG, Kyoto Encyclopedia of Genes and Genomes.
