# Supplementary material for: Senescence-related epicardial adipocyte genes lead to immune infiltration and myocardial infarction progression
Source: Front Cardiovasc Med. 2026 Mar 5;13:1759091. doi: 10.3389/fcvm.2026.1759091 (PMC12999425; doi:10.3389/fcvm.2026.1759091)
Supplement: Supplementary file 14 [file Table7.docx]

Supplementary Table 7. Immune cells related genes in pericardial layers.

| Macrophages | T cell CD4+ | Mast cells |
| --- | --- | --- |
| \| MMP12 \| \| --- \| \| LOC101928716 \| \| FDX1 \| \| CCND1 \| \| CD9 \| \| MMP7 \| \| GPNMB \| \| CAPG \| \| RNF128 \| \| CNIH3 \| \| DCSTAMP \| \| PAQR5 \| \| SLC2A5 \| \| ANO5 \| \| APOE \| \| SLC38A6 \| \| MREG \| \| SLC31A2 \| | \| CXCL13 \| \| --- \| \| CYFIP2 \| \| LEF1 \| \| NAPSB \| \| PRKXP1 \| \| LINC00926 \| \| ALOX5 \| \| SLC38A1 \| \| GPC3 \| \| CD3D \| \| MAL \| \| SORL1 \| \| LCK \| \| JAK3 \| \| MIR142 \| \| RAB37 \| \| EOMES \| \| P2RX5 \| \| IPCEF1 \| \| PRKCZ \| \| UBASH3A \| \| IKZF3 \| \| ITGB2-AS1 \| \| RASGRP1 \| \| NCF1 \| \| MAP4K1 \| \| RALGPS2 \| \| LBH \| \| FAM129C \| \| CD247 \| \| SOX8 \| \| CORO1A \| \| BEX2 \| \| FLJ38379 \| \| MYO1G \| \| ZC3H12D \| \| CD19 \| \| ZNF831 \| \| HMHA1 \| \| PIK3CD \| \| TSPAN5 \| \| RUNX3 \| \| CCL5 \| \| ZAP70 \| \| PNOC \| \| RASGRP2 \| \| HLA-DOB \| \| TBX21 \| \| RASSF5 \| \| PVRIG \| \| ICOS \| \| SPOCK2 \| \| TUBA4A \| \| CCL21 \| \| LAT \| \| CIITA \| \| CD79B \| \| CCR6 \| \| AFAP1L1 \| \| RASAL3 \| \| SNX20 \| | \| PCDH7 \| \| --- \| \| MYH11 \| \| COL8A1 \| \| CCL8 \| \| DKK2 \| \| GPC3 \| \| BRE-AS1 \| \| SORL1 \| \| DUSP2 \| \| NANOS1 \| \| HRASLS \| \| MAP7D2 \| \| FGF13 \| \| FOXC1 \| \| IRF1 \| \| TBX15 \| \| NBEA \| \| LBH \| \| EFCAB11 \| \| SOX8 \| \| STEAP1 \| \| MICAL2 \| \| AR \| \| PTGDS \| \| UGP2 \| \| C1orf53 \| \| PIK3CD \| \| LOC100287387 \| \| INMT \| \| S100B \| \| ACKR4 \| \| PHOSPHO2 \| \| CTSK \| \| OSMR \| \| HPGD \| \| SIDT1 \| \| IER3 \| \| ACOT13 \| \| SERPINE2 \| |
