# Supplementary material for: Senescence-related epicardial adipocyte genes lead to immune infiltration and myocardial infarction progression
Source: Front Cardiovasc Med. 2026 Mar 5;13:1759091. doi: 10.3389/fcvm.2026.1759091 (PMC12999425; doi:10.3389/fcvm.2026.1759091)
Supplement: Supplementary file 15 [file Table8.docx]

Supplementary Table 8. The methylation of the screened 82 overlapping genes.

| epi AND Gene.symbol NOT CAD | meth.diff | pvalue | CAD AND Gene.symbol NOT epi | meth.diff | pvalue |
| --- | --- | --- | --- | --- | --- |
| CISH | -7.950585999 | 0.033688252 | JAK2 | 11.76470588 | 0.029106804 |
| NKAPP1 | #N/A | #N/A | SIPA1L3 | 23.97183099 | 0.009055385 |
| ZSCAN30 | -18.94934334 | 0.044846454 | RABGEF1 | 15.64327485 | 0.021939904 |
| SLC38A5 | #N/A | #N/A | MS4A15 | -13.37719298 | 0.049730962 |
| SCAF11 | #N/A | #N/A | FGF12 | -7.317073171 | 0.035518 |
| XIRP2 | #N/A | #N/A | ADAM22 | 12.19512195 | 0.008757714 |
| LRP1 | -13.4502924 | 0.039764778 | CATSPER1 | 14.71339905 | 0.03801634 |
| KIAA1109 | #N/A | #N/A | ARSD | #N/A | #N/A |
| GNB4 | #N/A | #N/A | MTFR1 | 21.55172414 | 0.013922712 |
| HSD3B1 | #N/A | #N/A | ZNF99 | #N/A | #N/A |
| PTGS2 | #N/A | #N/A | PAX6 | 7.528735632 | 0.04240402 |
| DLG4 | -11.76470588 | 0.021558227 | OSTM1 | -7.407407407 | 0.049850296 |
| SMOX | 17.2979798 | 0.0107119 | SLC22A17 | 16.85435435 | 0.003604836 |
| CHRNA1 | #N/A | #N/A | SPP1 | #N/A | #N/A |
| HLA-DRA | #N/A | #N/A | NACA | #N/A | #N/A |
| MYBPC3 | 15.78947368 | 0.048722834 | GAP43 | #N/A | #N/A |
| EPHA3 | #N/A | #N/A | CENPI | #N/A | #N/A |
| MAPK8IP3 | -19.14285714 | 0.006403028 | SCN3A | #N/A | #N/A |
| ARSI | #N/A | #N/A | EPG5 | 16.11111111 | 0.049076234 |
| CXCL16 | -4.347826087 | 0.017614651 | NIN | -30.38961039 | 4.15407E-08 |
| USP9X | #N/A | #N/A | ELL2 | #N/A | #N/A |
| ASPH | -6.818181818 | 0.040543064 | SLC16A7 | -12 | 0.002972744 |
| CCDC88C | -13.48539416 | 0.039855369 | CFTR | #N/A | #N/A |
|  |  |  | CLK4 | #N/A | #N/A |
|  |  |  | IFI44L | #N/A | #N/A |
|  |  |  | ZDHHC18 | 22.25806452 | 0.018941672 |
|  |  |  | TMEFF2 | 2.272727273 | 0.04593325 |
|  |  |  | SPATA33 | #N/A | #N/A |
|  |  |  | IGFBP5 | -5.357142857 | 0.023924281 |
|  |  |  | RFX3 | #N/A | #N/A |
|  |  |  | CLDN11 | -11.42857143 | 0.028461363 |
|  |  |  | MAN1A2 | #N/A | #N/A |
|  |  |  | CDR1 | #N/A | #N/A |
|  |  |  | MUC15 | #N/A | #N/A |
|  |  |  | NMNAT2 | -7.317073171 | 0.02296501 |
|  |  |  | PIGQ | 12.10057622 | 0.037952041 |
|  |  |  | HDAC9 | 5.263157895 | 0.019713868 |
|  |  |  | PHGDH | 28.34054834 | 6.74342E-06 |
|  |  |  | CDKN2A | 12.5 | 0.007030921 |
|  |  |  | MLLT10 | 22.9978355 | 0.009367975 |
|  |  |  | PMEPA1 | 20.37037037 | 0.010959809 |
|  |  |  | GPR157 | -15.10416667 | 0.005172463 |
|  |  |  | TACC1 | -13.22997416 | 0.022371515 |
|  |  |  | VNN1 | #N/A | #N/A |
|  |  |  | EFNA5 | 6.25 | 0.04061903 |
|  |  |  | APLN | #N/A | #N/A |
|  |  |  | KRT222 | #N/A | #N/A |
|  |  |  | PRKCD | #N/A | #N/A |
|  |  |  | IRS2 | 8.333333333 | 0.01454706 |
|  |  |  | AKAP6 | #N/A | #N/A |
|  |  |  | MAP4K4 | #N/A | #N/A |
|  |  |  | KYNU | #N/A | #N/A |
|  |  |  | CCND2 | 4.838709677 | 0.048858471 |
|  |  |  | PITPNC1 | 13.79310345 | 0.021964315 |
|  |  |  | AOX1 | 3.614457831 | 0.047858017 |
|  |  |  | AK7 | 11.46153846 | 0.022677015 |
|  |  |  | PAOX | -14.52198467 | 0.046370023 |
|  |  |  | UBIAD1 | #N/A | #N/A |
|  |  |  | KIAA0895L | #N/A | #N/A |

CAD, coronary artery disease; epi, epicardial adipose tissue.
