# Supplementary material for: Senescence-related epicardial adipocyte genes lead to immune infiltration and myocardial infarction progression
Source: Front Cardiovasc Med. 2026 Mar 5;13:1759091. doi: 10.3389/fcvm.2026.1759091 (PMC12999425; doi:10.3389/fcvm.2026.1759091)
Supplement: Supplementary file 16 [file Table9.docx]

Supplementary Table 9. The baseline characteristics of patients in CAD and severe CAD group.

| Characteristic | Severe CAD | CAD | p |
| --- | --- | --- | --- |
| n | 8 | 4 |  |
| gender, n (%) |  |  | 0.208 |
| female | 4 (33.3%) | 0 (0%) |  |
| male | 4 (33.3%) | 4 (33.3%) |  |
| smoking, n (%) |  |  | 1.000 |
| 0 | 5 (41.7%) | 2 (16.7%) |  |
| 1 | 3 (25%) | 2 (16.7%) |  |
| nephropathy, n (%) |  |  | 1.000 |
| 0 | 6 (50%) | 3 (25%) |  |
| 1 | 2 (16.7%) | 1 (8.3%) |  |
| prior CAD, n (%) |  |  | 1.000 |
| 0 | 4 (33.3%) | 2 (16.7%) |  |
| 1 | 4 (33.3%) | 2 (16.7%) |  |
| high uric acid, n (%) |  |  | 1.000 |
| 0 | 5 (41.7%) | 2 (16.7%) |  |
| 1 | 3 (25%) | 2 (16.7%) |  |
| BMI, mean ± SD | 25.48 ± 4.3 | 23.78 ± 5.09 | 0.554 |
| Systolic pressure, mean ± SD | 129.25 ± 18.33 | 127 ± 8.12 | 0.823 |
| diastolic pressure, mean ± SD | 75.5 ± 12.72 | 81.75 ± 13.74 | 0.452 |
| LDL, mean ± SD | 2.87 ± 0.99 | 2.24 ± 0.56 | 0.270 |
| HDL, median (IQR) | 0.94 (0.82, 1) | 0.98 (0.94, 1.19) | 0.461 |
| CHO, mean ± SD | 4.55 ± 1.22 | 4.1 ± 0.66 | 0.512 |
| TG, mean ± SD | 1.61 ± 0.32 | 2.51 ± 1.05 | 0.184 |
| cTNI, median (IQR) | 0.68 (0.58, 9.23) | 0.74 (0.51, 1.25) | 0.799 |
| CK, median (IQR) | 83 (68.5, 223.5) | 105 (79.5, 126.25) | 0.734 |
| CK-MB, median (IQR) | 12 (11, 30.25) | 11.5 (9.75, 13.25) | 0.393 |
| BNP, median (IQR) | 166.75 (80.25, 664.92) | 78.9 (73.65, 346.8) | 0.552 |
