# Supplementary material for: Senescence-related epicardial adipocyte genes lead to immune infiltration and myocardial infarction progression
Source: Front Cardiovasc Med. 2026 Mar 5;13:1759091. doi: 10.3389/fcvm.2026.1759091 (PMC12999425; doi:10.3389/fcvm.2026.1759091)
Supplement: Supplementary file 17 [file Table10.docx]

Supplementary Table 10. The laboratory examinations at 1d after admission of patients in CAD and severe CAD group.

| Characteristic | severe CAD | CAD | p |
| --- | --- | --- | --- |
| n | 8 | 4 |  |
| acetone body, n (%) |  |  | 1.000 |
| 0 | 7 (58.3%) | 4 (33.3%) |  |
| 1+ | 1 (8.3%) | 0 (0%) |  |
| urine glucose, n (%) |  |  | 1.000 |
| 0 | 7 (58.3%) | 3 (25%) |  |
| 3+ | 1 (8.3%) | 1 (8.3%) |  |
| urine protein, n (%) |  |  | 1.000 |
| 0 | 6 (50%) | 4 (33.3%) |  |
| 1+ | 1 (8.3%) | 0 (0%) |  |
| 3+ | 1 (8.3%) | 0 (0%) |  |
| Src serum creatinine, median (IQR) | 74 (65.25, 103.75) | 83 (70.75, 220.5) | 0.734 |
| Fasting venous blood glucose, mean ± SD | 8.05 ± 3.76 | 6.94 ± 3.25 | 0.627 |
| glycosylated hemoglobin, median (IQR) | 6.65 (6.27, 8.4) | 6.35 (6.3, 7.35) | 0.797 |
| total bilirubin, mean ± SD | 11.91 ± 5.53 | 12 ± 5.75 | 0.979 |
| direct bilirubin, mean ± SD | 2.13 ± 1.68 | 2.68 ± 1.55 | 0.594 |
| indirect bilirubin, mean ± SD | 9.77 ± 6.49 | 9.31 ± 7.07 | 0.914 |
| alkaline phosphatase, mean ± SD | 80.5 ± 14.73 | 69.25 ± 13.33 | 0.228 |
| glutamic-pyruvic transaminase, median (IQR) | 38 (26.75, 51) | 30.5 (22.25, 46.75) | 0.808 |
| glutamic oxalacetic transaminase, median (IQR) | 27 (16.5, 67) | 21.5 (16.5, 29.25) | 0.570 |
| albumin, median (IQR) | 38.5 (37.3, 38.92) | 39.05 (36.5, 41.08) | 0.733 |
| globulin, mean ± SD | 22.56 ± 3.44 | 21.95 ± 1.69 | 0.748 |
