# Supplementary material for: Senescence-related epicardial adipocyte genes lead to immune infiltration and myocardial infarction progression
Source: Front Cardiovasc Med. 2026 Mar 5;13:1759091. doi: 10.3389/fcvm.2026.1759091 (PMC12999425; doi:10.3389/fcvm.2026.1759091)
Supplement: Supplementary file 18 [file Table11.docx]

Supplementary Table 11. The laboratory examinations at discharge after admission of patients in CAD and severe CAD group.

| Characteristic | severe CAD | CAD | p |
| --- | --- | --- | --- |
| n | 8 | 4 |  |
| urine protein, n (%) |  |  | 1.000 |
| 0 | 7 (58.3%) | 4 (33.3%) |  |
| 1+ | 1 (8.3%) | 0 (0%) |  |
| Src serum creatinine, median (IQR) | 77 (73.5, 118) | 79.5 (64.25, 107) | 0.704 |
| Fasting venous blood glucose, mean ± SD | 8.14 ± 1.65 | 5.86 ± 1.25 | 0.041 |
| total bilirubin, mean ± SD | 7.64 ± 1.67 | 13.68 ± 4.19 | 0.007 |
| direct bilirubin, mean ± SD | 2.34 ± 1.7 | 3.91 ± 2.89 | 0.278 |
| indirect bilirubin, mean ± SD | 5.3 ± 2 | 9.77 ± 4.86 | 0.162 |
| alkaline phosphatase, mean ± SD | 99.57 ± 27.67 | 112 ± 70.32 | 0.680 |
| glutamic-pyruvic transaminase, median (IQR) | 38 (23, 50.5) | 41 (24.5, 59.75) | 0.705 |
| glutamic oxalacetic transaminase, mean ± SD | 24.29 ± 12.58 | 27.25 ± 16.01 | 0.740 |
| albumin, median (IQR) | 36.5 (33.55, 37.3) | 37.3 (34.95, 38) | 0.633 |
| globulin, mean ± SD | 24.41 ± 3.67 | 19.52 ± 5.11 | 0.097 |
